# Peri-Operative Dosage and Therapeutic Concentrations of Cefazolin Administered for Surgical Site Infection Prophylaxis in Elective Surgery—A Systematic Review

**DOI:** 10.3390/antibiotics14121227

**Published:** 2025-12-05

**Authors:** Rochelle Ryan, Cemre Bosnak, Matthew Bright, Vesa Cheng, Gina Velli, Andre van Zundert, Jeffrey Lipman, Jason A. Roberts

**Affiliations:** 1 Department of Anaesthesia and Peri-Operative Medicine, Sunshine Coast Health, Birtinya, QLD 4575, Australia; 2 School of Medicine, The University of Queensland, Herston, QLD 4029, Australia; a.vanzundert@uq.edu.au; 3 Department of Infectious Diseases and Clinical Microbiology, Hacettepe University Hospital, Ankara 06230, Turkey; cemrebosnak@hacettepe.edu.tr; 4 Department of Anaesthesia, Princess Alexandra Hospital, Woolloongabba, QLD 4102, Australia; matthew.bright@health.qld.gov.au; 5 UQ Centre for Clinical Research, Faculty of Medicine, The University of Queensland, Herston, QLD 4029, Australia; v.cheng@uq.edu.au (V.C.); j.lipman@uq.edu.au (J.L.); jason.roberts@uq.edu.au (J.A.R.); 6 Medical Education Unit, Princess Alexandra Hospital, Woolloongabba, QLD 4102, Australia; 7 Department of Anaesthesia and Intensive Care, Faculty of Medicine, Chinese University of Hong Kong, Hong Kong 999077, China; 8 Library and Information Management, Princess Alexandra Hospital, Woolloongabba, QLD 4102, Australia; gina.velli@health.qld.gov.au; 9 Department of Anaesthesia and Peri-Operative Medicine, The Royal Brisbane and Women’s Hospital, Herston, QLD 4029, Australia; 10 UR UM 103, Division of Anesthesia, Critical Care and Emergency and Pain Medicine, Nimes University Hospital, University of Montpellier, 30029 Nimes, France; 11 Herston Infectious Disease Institute (HeIDI), Metro North Health, Herston, QLD 4029, Australia; 12 Departments of Intensive Care Medicine and Pharmacy, The Royal Brisbane and Women’s Hospital, Herston, QLD 4029, Australia

**Keywords:** surgical site infection, antibiotic prophylaxis, cefazolin, pharmacokinetics, systematic review, PRISMA

## Abstract

**Background/Objectives**: Cefazolin is commonly administered for surgical antibiotic prophylaxis. This review aims to examine whether target unbound plasma and tissue cefazolin concentrations are reached following prophylactic administration across multiple surgical subtypes. The primary outcome was a lower limit of cefazolin concentration variability (mean–SD, lower quartile, or lower range) in unbound plasma and/or tissue > 2 mg·L^−1^, the epidemiological cut-off (ECOFF) value for *Staphylococcus aureus* at skin incision and/or closure. **Methods**: Prisma 2020 guidelines were followed, and the protocol is registered in PROSPERO (CRD42021080289). A literature search using MEDLINE (PubMed), Embase, CENTRAL, CINAHL, and further databases was performed to identify studies in which prophylactic cefazolin was administered to adult surgical patients (≥18 years old) undergoing elective surgery, and unbound plasma and tissue concentrations were measured at skin incision and closure. Exclusion criteria included languages other than English, emergency surgery, cefazolin being administered for any reason other than surgical site prophylaxis, and whether patients received any cefazolin within the 48 h prior to the prophylactic dose. The search was repeated in August 2025 to ensure currency. A narrative assessment of the methodological quality was performed. The data were synthesised in a narrative and tabular form, and the certainty of the evidence was assessed using the GRADE approach. **Results**: A total of 37 studies with 1102 patients met the inclusion criteria. Twelve bariatric studies and 378 patients, 9 cardiac studies and 197 patients, 8 obstetric studies and 277 patients, 6 orthopaedic studies and 176 patients, 3 abdominal surgery studies and 62 patients, and 1 vascular study and 12 patients were included. Two studies met the inclusion criteria for both bariatric and abdominal surgery. The lower limit of variability of the unbound plasma concentration was consistently >2 mg·L^−1^. The reported lower limits of variability in tissue concentrations of bariatric surgery were conflicting. Only one study in cardiac surgery assessed the current dosing regimens. The lower range of variability of tissue concentrations was consistently >2 mg·L^−1^ in the orthopaedic, obstetric, abdominal, and vascular surgery subtypes. **Conclusions**: The current dosing approaches in the obstetric, orthopaedic, abdominal, and vascular surgery groups are reassuring for achieving effective concentrations, although the overall data are sparse. It is unclear if increased dosing is warranted in bariatric surgery patients, and further investigations in cardiac surgery with current dosing regimens are required.

## 1. Introduction

The prevalence of surgical site infections (SSIs) in Australia has been reported to be 3% [1], while the pooled 30-day cumulative incidence of SSIs may be as high as 11% worldwide [2]. Morbidity, mortality, and healthcare-associated costs increase with SSIs; therefore, the prevention of SSIs is imperative in peri-operative care. Effective antibiotic prophylaxis requires a consideration of the agent and dose, ensuring adequate drug concentrations in plasma and at the surgical site throughout the procedure. 

Cefazolin is the recommended antibiotic of choice for surgical prophylaxis [3,4] due to its safety and efficacy against common skin commensals that cause SSIs. The current guidelines recommend that 2 g of cefazolin is administered within 60 min of skin incision for adult patients <120 kg and that redosing be performed at two half-lives for prolonged procedures [3,4]. Achieving unbound cefazolin concentrations above 2 mg·L^−1^, the epidemiological cut-off (ECOFF) value for *Staphylococcus aureus* [5], during the operative period (skin incision to closure) in plasma and subcutaneous tissues can be considered the prophylactic target.

Quantifying the subcutaneous interstitial fluid (ISF) cefazolin concentration is essential, as this is the site most at risk for SSIs. Subcutaneous tissue concentrations can be measured via a homogenised adipose tissue analysis or microdialysis. When measuring antibiotic concentrations in homogenised adipose tissue samples, the resulting concentrations are the average of cefazolin concentrations in the ISF, adipose tissue cells, and blood capillaries, and may subsequently underestimate cefazolin ISF concentrations [6,7].

Microdialysis is an accepted and reliable method for measuring unbound cefazolin ISF concentrations. Researchers insert a small probe with a semipermeable membrane into the subcutaneous tissue. Unbound cefazolin concentrations are measured in the dialysate. A recovery ratio is calculated through the loss of an internal standard from the perfusate across the microdialysis membrane. This recovery ratio is then applied to determine the unbound ISF cefazolin concentration for the administered drug dose [8].

The optimal cefazolin dose for achieving adequate concentrations in the ISF of subcutaneous tissues and in plasma across various surgical patient groups remains undefined. The guidelines now recommend dose adjustments based on body size descriptors, recommending a 3 g dose if the total body weight (TBW) exceeds 120 kg [3,4] or 4 g if the body mass index (BMI) exceeds 35 kg·m^−2^ [9]. No other patient or surgical factors alter the recommended adult cefazolin dose in the current guidelines. The current inflexible dosing approach does not account for surgical and patient characteristics that may alter cefazolin pharmacokinetics and the subsequent probability of attaining target concentrations.

It is biologically plausible that patient factors, such as obesity and renal impairment, and surgical factors, such as cardiopulmonary bypass (CPB), may alter cefazolin pharmacokinetics and impact the achievement of target tissue concentrations. Several small pharmacokinetic studies have examined cefazolin target attainment in plasma and/or subcutaneous tissue. To the best of our knowledge, no systematic reviews have examined whether these dosing recommendations are sufficient for achieving therapeutic concentrations in plasma and tissues across different surgical subtypes.

This systematic review aims to examine whether unbound plasma and/or tissue concentrations following prophylactic cefazolin regimens administered to adult, elective surgical patients achieve acceptable concentrations across different surgical subtypes. The primary outcome measure is the achievement of a lower limit of variability (first quartile, mean–SD, or lower range) in unbound plasma and/or a subcutaneous tissue concentration above 2 mcg·g^−1^ if measured using the homogenised tissue concentration, or an ISF concentration above 2 mg·L^−1^ if measured using microdialysis, at skin incision and/or skin closure in each different surgical subspecialty.

## 2. Results

### 2.1. Search Results

**Figure 1:**
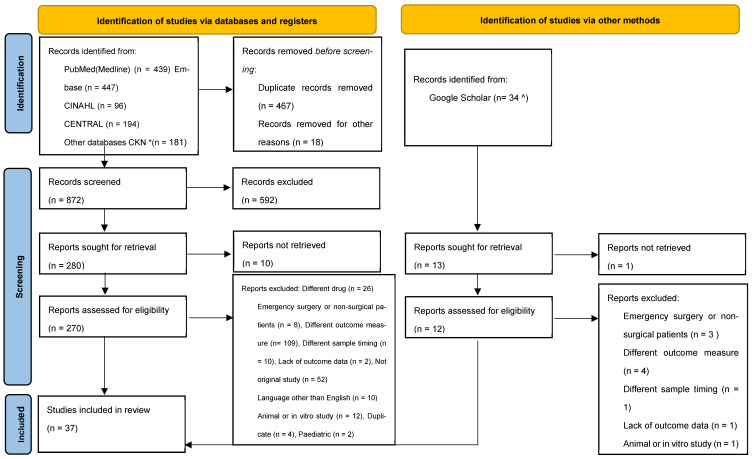
PRISMA flow diagram for the systematic review detailing the database searches, the number of articles screened, full texts retrieved, and the number of studies included. * Strategy adapted for Clinical Knowledge Network EBSCO EDS discovery layer and run across multiple additional databases; full list in Supplementary Materials. ^ Google Scholar; 21 duplicates screened out.

Figure 1 presents the PRISMA flow diagram. The initial search across all databases yielded a total result of 1391 publications, of which 488 were duplicates and 18 were duplicate English translations, and 594 records screened were not suitable for inclusion. A total of 37 studies, which include 1102 patients, matched the criteria for the paper review. Two of the papers met the criteria for both bariatric surgery and abdominal surgery [10,11]. The reviewers further assessed selected publications for any further published notifications of “errata”, “retractions”, “errors”, and “omissions”.

### 2.2. Bariatric Surgery

Twelve studies [10,11,12,13,14,15,16,17,18,19,20,21] measured unbound plasma and/or tissue concentration at skin incision and/or closure in patients undergoing bariatric surgery and included 378 patients (Table 1).

#### 2.2.1. Unbound Plasma Cefazolin Concentrations in Bariatric Surgery

Seven studies measured unbound plasma concentrations. Six studies had a protocol which included a 2 g dose [10,11,13,14,16,17]. For unbound plasma, the lower limits of variability were above 2 mg·L^−1^ at skin incision in all studies. At skin closure [15] and up to 240 min [11,13,16], 300 min [17], and 480 min [10] after cefazolin administration, the unbound plasma cefazolin concentration lower limits of variability were all above 2 mg·L^−1^. One study compared a 2 g and 3 g dose in patients with mean BMIs of 49.0 ± 5.4 and 44.0 ± 5.1 kg·m^2^, respectively [16]; both groups achieved a lower limit of variability in plasma of approximately 7 and 10 mg·L^−1^, respectively, at 240 min after administration.

Four studies compared cefazolin concentrations across two cohorts of different body sizes [10,11,14,15]. Brill et al. [11] and Dorn et al. [10] compared cefazolin pharmacokinetics in obese and non-obese patients. Brill et al. [11] found that a 2 g dose administered in the non-obese patients undergoing laparoscopic fundoplication (mean ± standard deviation (SD) BMI; 28.2 ± 5 kg·m^−2^) and obese patients undergoing laparoscopic gastric bypass surgery (mean ± SD; BMI 47 ± 6) resulted in a limit of variability (lower quartile range) > 2 mg·L^−1^ in both groups. There was no significant difference in the area under the curve (ƒAUC_0–4h_) between the two groups (*p* > 0.05). Dorn et al. [10] administered 2 g of cefazolin to bariatric patients and non-obese patients (BMI median (min-max); 51.7 (39.5–69.3) vs. 26.0 (18.7–29.8) kg·m^−2^, respectively) undergoing abdominal surgery. The lower limit of variability (mean–SD) was >2 mg·L^−1^ at 60 and 240 min after cefazolin administration in both groups. However, the maximum concentration and ƒAUC_≈_ were significantly different between the obese and non-obese group (median (min–max) BMI; 51.7 (39.5–69.3) vs. 26.0 (18.7–29.8) kg·m^−2^).

Hites et al. [14] and Cincotti et al. [15] described cefazolin pharmacokinetics across different body sizes within the bariatric population. The study by Hites et al. [14] included 12 patients having non-bariatric abdominal surgery in the <35 kg·m^−2^ group. The lower limit of variability (mean–SD) was >2 mg·L^−1^ in subjects with BMI > 35 kg·m^−2^ and subjects with BMI < 35 kg·m^−2^ at 30 min after cefazolin administration. The lower limit of variability (mean–SD) was >2 mg·L^−1^ at 180 min for both groups but was difficult to discern from the graph at 240 min. Cincotti et al. [15] administered 4 g of cefazolin to patients undergoing sleeve gastrectomy. The mean unbound concentrations were significantly higher in the patient groups with a mean BMI 44 ± 2.5 kg·m^−2^ compared to the group with a mean BMI 54.3 ± 4 kg·m^−2^ (*p* < 0.0001, two-way analysis of variance). The lower limit of variability for unbound plasma concentrations in both groups, however, remained >10 mg·L^−1^ at the skin incision and closure.

Across the available studies, the reported lower limit of unbound concentration variability in plasma following 2 g of cefazolin was >2 mg·L^−1^ in patients undergoing bariatric surgery. A cefazolin 2 g dose achieved adequate unbound plasma concentrations in bariatric surgical patients.

#### 2.2.2. Subcutaneous Tissue Concentrations in Bariatric Surgery

Ten studies [10,11,12,15,16,17,18,19,20,21] measured the tissue concentrations of cefazolin in patients undergoing bariatric surgery, four of whom used microdialysis. Following a 2 g bolus, the lower limit of variability in the ISF concentration was >2 mg·L^−1^ at incision [10,11,16,17], 240 [16], 270 [17], and 480 min [10] after cefazolin administration. In the study by Brill et al. [11], it is difficult to discern from the graph the lower limit of variability in the obese group (BMI mean ± SD; 47.0 ± 5.8 kg·m^−2^), however, it is likely >2 mg·L^−1^ at 120 min.

Dorn et al. [10] and Brill et al. [11] measured ISF concentrations using microdialysis across two cohorts of different body sizes having bariatric and non-bariatric surgery. In the study by Dorn et al. [10], thirty minutes following a 2 g cefazolin dose, the lower limits of variability in ISF concentrations (mean–SD) were approximately 8 and 16 mg·L^−1^ in obese and non-obese groups (having non-bariatric abdominal surgery; also see abdominal surgery section), respectively (median (min–max) BMI; 51.7 (39.5–69.3) vs. 26.0 (18.7–29.8) kg·m^−2^), and approximately 4 mg·L^−1^ in both groups at 270 min. The median (range) surgical duration was 162 (84–234) and 162 (50–480) in the non-obese and obese groups. At 480 min, the lower limits of variability (mean–SD) were 2 mg·L^−1^ and approximately 1.5 mg·L^−1^, indicating that, for prolonged surgeries, the ISF is likely to be below the acceptable target in a proportion of patients.

Brill et al. [11] determined a lower quartile of approximately 20 mg·L^−1^ at 30 min after a 2 g dose in obese patients undergoing bariatric surgery and non-obese patients undergoing laparoscopic fundoplication surgery (mean ± SD BMI; 47 ± 6 vs. 28.2 ± 5 kg·m^−2^). The lower quartile concentrations were >5 mg·L^−1^ in the non-obese group, and, although difficult to discern in the obese group, it is likely > 2 mg·L^−1^ at 120 min. One study [16] used a 2 or 3 g intravenous cefazolin dose and measured the ISF concentration using microdialysis. The lower limits of variability in ISF concentrations were >7 mg·L^−1^ at 20 and 240 min in patients who received both 2 and 3 g of cefazolin (mean ± SD BMI; 49.7 ± 5.4 and 44.0 ± 5.1 kg·m^−2^). In another study [17], 2 g of cefazolin was administered at induction to 14 patients (mean ± SD BMI; 50.0 ± 11.2 5.1 kg·m^−2^) undergoing bariatric surgery. The lower limit of variability in ISF concentrations was approximately 3 at 180 min but was <2 mg·L^−1^ at 2 and 4 h, indicating a high variability, potential measurement bias, and that some patients had concentrations < 2 mg·L^−1^ during the surgical period.

Six studies measured homogenised adipose tissue cefazolin concentrations, with mixed results [12,15,18,19,20,21]. Forse et al. [19] described concentrations following a 1 g intravenous dose administered 12–15 min before skin incision. Participants in the normal weight group (mean ± SD BMI 22 ± 4 kg·m^−2^) and morbidly obese group (BMI 47 ± 6 kg·m^−2^) had lower limit of variability (mean–SD) concentrations of 4.7 and 1.7 mcg·g^−1^, respectively, at incision and 3.3 and 1.4 mcg·g^−1^ at closure, respectively. A 1 g dose 2 h before induction and a further 1 g at induction resulted in incision and closure lower limits of variability of 3.0 and 1.9 mcg·g^−1^ in 13 patients with a mean ± SD TBW of 128.8 ± 20.0 kg·m^−2^. A standard 2 g dose 15 min before incision resulted in a lower limit of variability (mean–SD) at incision of 3.7 mcg·g^−1^ in 37 participants with a mean ± SD BMI of 46 ± 8 kg·m^−2^ [21]. The closure lower limits of variability were 2.7, 3.1, and 3.7 mcg·g^−1^ in participants who underwent laparoscopic sleeve gastrectomy, laparoscopic sleeve gastrectomy plus another procedure, and Roux-en-Y gastric bypass, respectively.

Intravenous cefazolin doses greater than 2 g yielded conflicting results. Edmiston et al. [12] found a lower limit of variability > 2 mg·L^−1^ at incision following a 2 g intravenous dose before the induction of anaesthesia in three groups with mean ± SD BMIs of 47.0 ± 1.3, 53.9 ± 2.8, and 69.2 ± 10 kg·m^−2^ (mean–SD of 1.9, 0.8, and 1.0 mcg·g^−1^, respectively). A second dose was 2 g of cefazolin given 3 h after the initial dose. In the group who had the lowest BMI (mean), the lower limit of variability at closure was 2.5 mcg·g^−1^; however, in the two higher BMI groups, the results were 0.7 and 1.5 mcg·g^−1^. The surgical durations (mean) were the longest in this study compared to the other bariatric surgery studies, and the BMIs (means) were also high.

A 2 g bolus at induction followed by an infusion of 1 g over 2 h resulted in incision and closure lower limits of variability of 4.1 mcg·g^−1^ and 5.4 mcg·g^−1^ in 18 participants with a BMI mean ± SD of 40.6 ± 4.0 kg·m^−2^ [20]. A 4 g dose resulted in adequate lower limit of variability concentrations of >2 mg·L^−1^ at incision and closure [15], including in the higher BMI group (mean ± SD BMI 54.3 ± 4.1 kg·m^−2^).

In most studies, a 2 g bolus of cefazolin administered to bariatric surgical patients as a surgical prophylaxis achieves a lower limit of variability of tissue concentration that is >2 mg·L^−1^ for the duration of surgery. A 2 g cefazolin bolus administered to bariatric surgical patients is highly likely to achieve satisfactory tissue concentrations.

### 2.3. Cardiothoracic Surgery

Nine studies examine unbound plasma cefazolin concentrations or subcutaneous adipose tissue concentrations in 197 patients undergoing cardiothoracic surgery (Table 2).

#### 2.3.1. Unbound Plasma Cefazolin Concentrations in Cardiac Surgery

Five studies [23,25,27,28,30] measured unbound plasma cefazolin concentrations. Four of the studies [25,27,28,30] demonstrated a lower limit of variability for unbound cefazolin concentrations > 2 mg·L^−1^ at skin incision and closure.

Hollis et al. [25] administered 2 g of cefazolin intravenously within 1 h of incision and then 2 g every 3 h into the CPB circuit in 10 patients (mean ± SD TBW 85 ± 28 kg). The unbound plasma concentration lower limits of variability (mean–SD) were 31 and 23 mg·L^−1^ at skin incision and closure, respectively. Similarly, the lower limits of variability (mean–SD) were >50 mg·L^−1^ and 20 mg·L^−1^ at 0.5 and 3.5 h, respectively, following a 2 g dose in 16 patients undergoing valve surgery with a median ± SD age of 44.0 ± 12.0 years and a BMI of 28.2 ± 6.7 kg·m^−2^ [30]. Following 1 g before incision and then 1 g every four hours, the lower limit of variability (lower range) concentration was 11 and 21 mg·L^−1^ at incision and closure, respectively, in 27 patients (mean TBW 62 ± 12 kg) [27]. In a further study of 40 patients, a mean (SD) cefazolin dose of 23.5 ± 5.4 mg·kg^−1^ was administered before skin incision [28]; the lower limit of variability (mean–SD) for the unbound cefazolin concentration at closure was 6.6 mg·L^−1^.

Kosaka et al. [23] gave 62 patients with varied renal function a 2 g intravenous dose before skin incision, followed by 1 g every 6 h, and a further 1 g in the priming circuit if CPB was used. The lower limit of variability (mean–SD) for unbound cefazolin concentration at skin incision and closure was above 2 mg·L^−1^ in all patient groups. The lower limit of variability was >20, 30, and 50 mg·L^−1^ in patients with a creatinine clearance > 50 mL·min^−1^, between 10 and 49 mL·min^−1^, and on dialysis, respectively. The closure lower limit of variability was difficult to discern from the graph in patients with a creatinine clearance > 50 mL·min^−1^; however, it was likely >2 mg·L^−1^. The closure lower limits of variability were approximately 30 and 60 mg·L^−1^ in patients with a creatinine clearance between 10 and 49 mL·min^−1^ and on dialysis, respectively.

In the included studies, cefazolin 2 g administered prior to cardiac surgery achieves a lower limit of variability of an unbound plasma concentration of >2 mg·L^−1^. Given the overall large effect size demonstrated in these studies, cefazolin 2 g achieves the target prophylactic unbound plasma concentrations in cardiac surgical patients.

#### 2.3.2. Subcutaneous Tissue Concentrations in Cardiac Surgery

Subcutaneous adipose tissue concentrations were measured in four studies [24,26,27,29], and the results are conflicting.

Tchaick et al. [26] measured tissue concentrations using homogenised adipose tissue [26]. The homogenised adipose tissue lower limit of variability was 3.9 mcg·g^−1^ and 5.5 mcg·g^−1^ at skin incision and closure, respectively, after 19 patients received a 2 g bolus at induction, followed by 1 g every 4 h [26].

Three studies used microdialysis [22,24,29]. Higher dosing regimens were administered compared to Tchaick et al. [26]; however, the tissue lower limit of variability was below the target concentrations at either 20 min [22] or 600 min [24,29] in these studies.

Following a 4 g intravenous dose 60 min before skin incision and a 2 g intravenous dose at skin closure (mean ± SD surgical duration: 188 ± 39 min), the lower limit of variability (mean–SD) for ISF concentrations, measured using microdialysis, was approximately 20 mg·L^−1^ at 60 min in seven patients (mean ± SD age, 30 ± 13 years; and mean ± SD TBW, 76 ± 12 kg) [22]. At 250 min after the first dose, the lower limit of variability was reported as >20 mg·L^−1^.

Andreas et al. [24] reported an ISF lower limit of variability (mean–SD) of >15 mg·L^−1^ (right side) and 7.3 mg·L^−1^ (left side) at incision following a 4 g dose before incision. A further 2 g was given approximately 1 h before closure, and the closure lower limit of variability was 5 mg·L^−1^ and 0.2 mg·L^−1^ at 600 min. The surgical duration was not recorded. The lower limit of variability was <2 mg·L^−1^ at 300 min for the left side and for both sides at 480 and 540 min. To improve the tissue exposure, the study was repeated [29] with a regimen of 4 g at 3 h and 1 h pre-incision and an additional 2 g at closure. The lower limit of variability (mean–SD) was 8.1 mg·L^−1^ (right sternal side) and 8.6 mg·L^−1^ (left sternal side) at incision but remained <2 mg·L^−1^ (left side) at 540 and 600 min. Furthermore, the mean percentage the free target concentration was above 2 mg·L^−1^ was 0.9 ± 0.2 for 0–10 h on the left side, indicating that the target concentrations were not reached for the duration of the study period despite the higher dosing.

Whilst the results of Tchaick et al. [26] are reassuring, showing that a 2 g cefazolin dose and redosing at 4 h achieves the target lower limit of variability of tissue concentration, there are limited studies using the currently recommended dosing regimens. Additionally, the results of Andreas et al. [24] raise concerns that a measurement bias in the context of left mammary artery harvesting may exist. Further investigation is required. There is currently insufficient evidence to determine if cefazolin 2 g achieves a lower limit of variability of tissue concentrations > 2 mg·L^−1^ in cardiac surgical patients.

### 2.4. Caesarean Delivery

Eight studies reported subcutaneous adipose tissue cefazolin concentrations in 277 patients undergoing caesarean delivery (CD) (Table 3) [31,32,33,34,35,36,37,38]. Only Eley et al. [37] measured unbound plasma cefazolin concentrations.

#### 2.4.1. Unbound Plasma Concentrations in Caesarean Delivery

Unbound plasma concentrations were measured by Eley et al. [37] in 12 obese women (mean ± SD weight; 119.1 ± 18.8 kg) undergoing CD who received a 2 g cefazolin bolus within 30 min of skin incision. The longest surgical duration was 125 min (mean ± SD; 75.8 ± 21.0). The lower limit of variability (median–SD) was >2 mg·L^−1^ at 30 and 180 min.

Given the effect size, it is reassuring that a 2 g intravenous cefazolin bolus administered prior to CD is highly likely to achieve an effective unbound plasma concentration, even in the obese population.

#### 2.4.2. Subcutaneous Tissue Concentrations in Caesarean Delivery

Eley et al. [37] also measured subcutaneous tissue concentrations using microdialysis in the aforementioned study. The lower limit of variability (median–SD) was difficult to determine from the graph but is likely >2 mg·L^−1^ at 30 and 120 min (longest surgical duration 120 min). Two subjects did not maintain concentrations > 2 mg·L^−1^ for the duration of the sampling period.

In the study by Dotters-Katz et al. [38] the lower quartile was >2 mcg·g^−1^ at closure in the group with the lower blood loss. The authors sought to determine if quantitative blood loss (QBL) during an elective caesarean section would alter adipose tissue concentrations. The low QBL group was defined as QBL > the 75th percentile, while the high QBL group was defined as QBL > the 75th percentile of the study population. Following a 2 g intravenous cefazolin bolus within 60 min of skin incision, the lower quartile tissue concentrations were >2 mcg·g^−1^ in both groups (low QBL group: 4.1 mcg·g^−1^; high QBL group: 3.6, 9.7 mcg·g^−1^). The closure lower quartile concentrations were 1.9 in the low QBL group, but 3.0 in the high QBL group.

Researchers in three randomised controlled trials (RCTs) [31,32,33] measured homogenised adipose tissue concentrations after different cefazolin doses were administered to obese women undergoing CD. All groups had a lower limit of variability tissue concentration > 2 mcg·g^−1^ at incision and closure. Two studies compared a 2 g and 3 g intravenous dose. A 2 g dose in 28 women with a median interquartile range (IQR) BMI of 38.9 (35.4–45.6) kg·m^−2^ resulted in a lower limit of variability (lower quartile) of 5.1 mcg·g^−1^ and 4.4 mcg·g^−1^ at incision and closure, respectively [32]. The comparison group of 30 women with a median (IQR) BMI of 39.3 (36.7–44.8) kg·m^−2^ was administered 3 g of cefazolin and had a lower quartile concentration of 7.0 mcg·g^−1^ and 6.7 mcg·g^−1^. Similarly, Young et al. [33] found that the lower quartile concentration was 5.4 mcg·g^−1^ and 9.2 mcg·g^−1^ at incision and closure, respectively, in 13 women who received a 2 g intravenous dose. This was compared to 13 women who had a lower quartile concentration of 5.4 mcg·g^−1^ and 10.8 mcg·g^−1^ at incision and closure, respectively, after a 3 g dose.

Stitely et al. [31] found a lower limit of variability (mean–SD) above 2 mcg·g^−1^ at incision and closure after both 2 g (*n* = 11) and 4 g intravenous doses (*n* = 9), with a lower limit of variability at incision of 11.7 vs. 16.0 mcg·g^−1^ and closure of 15.7 vs. 17.5 mcg·g^−1^ after 2 g and 4 g doses, respectively.

Three prospective observational studies compared two cohorts of different body sizes [34,35,36]. All had a lower limit of variability of >2 mcg·g^−1^ at incision and closure. Pevzner et al. [34] found mean incision concentrations following a 2 g intravenous dose higher in non-obese participants with a BMI < 30 kg·m^−2^ (mean ± SD BMI; 26.7 ± 1.3 kg·m^−2^) compared with obese participants with a BMI 30–40 kg·m^−2^ (mean ± SD BMI; of 34.1 ± 2.6 kg·m^−2^) (*p* = 0.009) or extremely obese participants with BMI > 40 kg·m^−2^ (mean ± SD BMI; 44.5 ± 4.5 kg·m^−2^) (*p* < 0.001); however, the lower limit of variability (mean–SD) was >2 mcg·g^−1^ in all groups at incision and closure (6.7 and 2.7 mcg·g^−1^ vs. 4.1 and 3.1 mcg·g^−1^ vs. 3.2 and 3.2 mcg·g^−1^, respectively). Swank et al. [35] then recruited a further 14 women with a BMI of 30–40 kg·m^−2^ and 14 women with a BMI > 40 kg·m^−2^ and administered 3 g intravenously to each participant. The lower quartiles of variability were 20.3 mcg·g^−1^ and 7.6 mcg·g^−1^ at incision and 18.9 mcg·g^−1^ and 6.2 mcg·g^−1^ at closure in each group, respectively (Mean ± SD BMI: 33.8 ± 2.9 vs. 45.0 ± 3.8 kg·m^−2^). These data were then compared to the patients in Pevzner et al., who received 2 g doses [34]. The concentrations were significantly higher in the 3 g group compared to the 2 g group with the same BMI range.

Kram et al. [36] administered 2 g intravenously to patients weighing >120 kg, and 3 g intravenously to patients weighing >120 kg. The lower quartile concentrations were 3.0 mcg·g^−1^ and 2.8 mcg·g^−1^ in the 2 g group and 3.9 mcg·g^−1^ and 2.6 mcg·g^−1^ in the 3 g group at incision and closure, respectively.

These data support that cefazolin 2 g will achieve a lower limit of variability of tissue concentration > 2 mg·L^−1^ in patients undergoing CD.

### 2.5. Orthopaedic and Spinal Surgery

Six studies in orthopaedic surgery [1,39,40,41,42,43] and 176 patients were included (Table 4). Two studies have described unbound plasma cefazolin concentrations in orthopaedic surgeries [39,40], while four studies have examined subcutaneous adipose tissue concentrations in this patient group [41,42,43,44].

#### 2.5.1. Unbound Plasma Concentrations in Orthopaedic and Spinal Surgery

Naik et al. [39], randomised 20 patients to receive either (1) an intermittent bolus regimen of 2 g intravenously 15–60 min before incision, followed by 2 g at 4 h; or (2) a 2 g intravenous bolus 15–60 min before incision, followed by a continuous infusion of 500 mg·h^−1^ for the duration of the procedure. Thirty minutes after the initial bolus, the intermittent group had an unbound plasma concentration lower limit of variability (mean–SD) of approximately 15 mg·L^−1^ (mean ± SD BMI; 27 ± 5 kg·m^−2^), and the continuous infusion group (mean ± SD BMI; 27 ± 4 kg·m^−2^) had a lower limit of variability concentration of >30 mg·L^−1^. This study included patients undergoing urological surgery, which therefore reduces the external validity.

In a study of 46 patients (mean ± SD BMI; 29.8 ± 7.5) undergoing orthopaedic, spinal, and abdominal surgery who were given 1 g before surgery (7 patients’ dosing varied from this protocol) the lower limit of variability (mean–SD) unbound plasma concentration was 2.8 mg·L^−1^ at closure [40]. The addition of 15 patients undergoing abdominal surgery in this study affects the confidence in applying the results to the orthopaedic and spinal surgical groups.

Whilst the lower limit of variability of plasma concentration reported in these studies is reassuring, due to the small number of studies and reduced external validity, there is insufficient evidence to determine if cefazolin 2 g is sufficient to achieve the target prophylactic concentrations in patients undergoing orthopaedic surgery.

#### 2.5.2. Subcutaneous Tissue Concentrations in Orthopaedic and Spinal Surgery

Four studies examined subcutaneous homogenised adipose tissue concentrations [41,42,43,44]. All four studies had lower limits of variability > 2 mcg·g^−1^. Thirty patients (mean ± SD BMI; 25.6 ± 2.7 kg·m^−2^) [42] received 2 g of cefazolin 30 min before tourniquet inflation; the lower limit of variability (mean–SD) homogenised adipose tissue concentration was reported to be 9.5 mcg·g^−1^ at skin incision. Similarly, in a study that was designed to validate a novel method of liquid chromatography–tandem mass spectrometry, the subcutaneous adipose tissue concentration lower limit of variability (mean–SD) at incision was 4.0 mcg·g^−1^ when 2 g of cefazolin was given at least 10 min before tourniquet inflation or skin incision [44]. However, demographic data were not available. Another study administered a 1 g intravenous bolus of cefazolin in 11 patients, with a mean (range) BMI of 65.3 (48–83) kg·m^−2^, 10–30 min before tourniquet inflation [41]. Despite a lower than recommended dose, the incision and closure lower limits of variability (mean–SD) were 2.9 mcg·g^−1^ and 5.1 mcg·g^−1^, respectively.

The relative timing of tourniquet inflation to antibiotic administration may impact the delivery of antibiotics to the surgical site. Montreuil et al. [43] administered 2 g of intravenous cefazolin within 60 min of skin incision. In total, 29 out of 59 patients had a tourniquet inflated. The incision and closure homogenised adipose tissue concentration lower limits of variability (lower 95% CI) were 5.0 mcg·g^−1^ and 6.3 mcg·g^−1^, respectively. The adipose tissue concentration lower limits of variability in 30 patients in the non-tourniquet group had concentrations of 6.3 mcg·g^−1^ and 7.0 mcg·g^−1^ at 90 min (mean ± SD duration of surgery was 77.8 ± 16.7 min and 81.4 ± 19.0 min in the tourniquet and non-tourniquet groups, respectively).

Whilst the study numbers are small, a 2 g prophylactic dose of cefazolin in orthopaedic surgical patients achieves a lower limit of variability of tissue concentration > 2 mg·L^−1^.

### 2.6. Abdominal Surgery

Three studies [10,11,45] in 62 patients described unbound cefazolin plasma concentrations in patients undergoing non-bariatric gastrointestinal surgery, and two of these studies [10,11] examined subcutaneous adipose tissue concentrations (Table 5). 

#### 2.6.1. Unbound Plasma Concentrations in Abdominal Surgery

Kim et al. [45] described unbound cefazolin plasma concentration in 40 patients (mean ± SD TBW; 67 ± 13 kg) undergoing gastric surgery. Patients received cefazolin 2 g, infused over 10 min, before skin incision. Mean or median concentrations were not presented; however, at 120 min, the concentrations were all >3 mg·L^−1^. At 360 min, there was a large proportion of patients with concentrations > 2 mg·L^−1^. The mean ± SD surgical duration was 142.0 ± 40.7 min.

In a study by Dorn et al. [10], cefazolin 2 g was administered as an infusion over a median (range) duration of 25 (21–30) min to 15 patients (median (range) age; 45 (21–65) years, median (range) BMI; 26.0 (18.7–29.8) kg·m^−2^) undergoing abdominal or pelvic surgery. Surgery types included tumour resections of the liver, pancreas, colon, or cervix, rection of cysts on the liver or ovaries, uterus myomatosus, cholecystectomy, hernia repair, and achalasia surgery. The infusion was started at a median (range) of 30 (3–71) minutes before skin incision in all patients. The median (range) of surgical duration was 162 (50–480) mins), with the unbound cefazolin concentration lower limit of variability (mean–SD) reported to be >25 mg·L^−1^ and approximately 6 mg·L^−1^ at 30 and 240 min, respectively.

Seven patients (mean ± SD BMI; 28.2 ± 2.8 kg·m^−2^) undergoing laparoscopic fundoplication were administered 2 g of cefazolin approximately 15 min before incision [11]. The lower limits of variability (lower quartile) in unbound plasma concentrations were approximately 30 mg·L^−1^ and >15 mg·L^−1^ at 30 and 120 min, respectively.

Given the large effect size observed in these studies, cefazolin 2 g consistently achieves a lower limit of variability of unbound plasma concentration > 2 mg·L^−1^ in patients undergoing abdominal surgery.

#### 2.6.2. Subcutaneous Tissue Concentrations in Abdominal Surgery

Dorn et al. [10] also measured ISF concentrations using microdialysis. The ISF concentration lower limits of variability were reported to be approximately 4 mg·L^−1^ and 7 mg·L^−1^ in the 0–30 min and 180–240 min sampling periods, respectively. The lower limits of variability (lower quartile) measured in the study by Brill et al. [11] were approximately 20 mg·L^−1^ and >5 mg·L^−1^ at 20 and 120 min, respectively.

Although there is a limited number of included patients in these two studies (*n* = 22), the data supports that cefazolin 2 g achieves a lower limit of variability of tissue concentration > 2 mg·L^−1^.

### 2.7. Vascular Surgery

One study described unbound plasma and subcutaneous adipose tissue concentrations in vascular surgery patients [46] (Table 5). Twelve patients undergoing elective or semi-elective abdominal aortic aneurysm repair were administered cefazolin 2 g 30 min before incision. The unbound plasma concentration lower limit of variability (first quartile) was >10 mg·L^−1^ and approximately 8 mg·L^−1^ at 30 and 200 min, respectively. The ISF concentration lower limits of variability were >2 mg·L^−1^ and 10 mg·L^−1^ at 30 and 200 min, respectively.

Although only one small study, the data support that, following cefazolin 2 g, the lower limit of variability of both unbound plasma and tissue concentrations is >2 mg·L^−1^ in patients undergoing vascular surgery.

### 2.8. Quality Assessment, Summary of Findings, and Certainty of Evidence

A narrative risk of bias assessment of individual studies is presented in Supplementary Material Table S1.

The certainty of evidence ranged from very low to high (Table 6). Commonly, the certainty of evidence for plasma data was higher than the tissue data due to the larger effect size observed in the plasma compared to the tissue. The certainty of evidence was commonly downgraded due to the small number of included studies, which included patients introducing risk of a bias, inconsistency in results, and also indirectness when external validity was reduced due to the inclusion of other surgical population groups in the studies.

## 3. Discussion

All studies demonstrated that a 2 g cefazolin dose achieves a lower limit of variability in unbound plasma concentrations > 2 mg·L^−1^ across all surgical subtypes. The more relevant compartment in SSIs is the tissue compartment, and the achievement of a lower limit of variability in tissue concentration was variable.

The necessity to increase doses for patients weighing more than 120 kg [3,4] or with a BMI > 35 kg·m^−2^ [9] is unclear. Researchers have found a negative correlation between body size and both tissue concentrations [11,14,15] and tissue penetration [11,47]. Some studies in patients undergoing bariatric surgery demonstrated that the lower limit of variability in tissue was >2 mg·L^−1^ at incision and closure following a 2 g dose in some [10,16,19,20,21], but not all, studies [12].

The results in other bariatric surgery studies are unclear. Ryan et al. [17] demonstrated a lower limit of variability of >2 mg·L^−1^ at 30 and 180 min, but not 1at 20 and 240 min, indicating a high variability in tissue concentrations and that some subjects did not achieve target tissue concentrations for the study period. At 120 min, the lower range of variability in tissue concentrations was challenging to determine in the study by Brill et al. [11]. But certainly some subjects had concentrations below the target threshold during the sampling period, as evidenced by the observed vs. predicted plots. When a 3 g [16] or a 4 g dose [15] was administered, the lower limits of variability in tissue were acceptable at incision and closure.

Monte Carlo simulations were performed in three bariatric surgery studies and indicate that achieving a target of 2 mg·L^−1^ for up to 3 h is achievable with a 2 g dose [11,16,17]. This suggests that 2 g is likely to be an adequate dose to achieve the target concentration. However, robust randomised controlled trials comparing 2 g with higher dosing regimens are needed to establish dose adequacy in bariatric surgery patients.

Researchers used varying dosing regimens in the cardiothoracic surgery population. Guidelines recommend a single 2 g cefazolin dose 60 min before incision, with redosing in two half-lives [3,4], without alterations for CPB. The necessity for prolonged 24–48 h prophylactic antibiotics is debated [48,49] and under further investigation [50]. The lower limits of variability tissue concentrations were greater than the target at incision with standard [26] and increased [22,24,29] dosing regimens.

Andreas et al. have demonstrated in two studies [24,29] that left internal mammary artery harvesting reduces tissue penetration on the left side, and the subsequent left-sided lower limit of variability tissue concentrations were <2 mg·L^−1^ during the prolonged sampling period, even with an increased dosing regimen. Andreas et al. [24] did not state the surgical duration, and therefore it cannot be determined if the target tissue concentrations were maintained throughout surgery. Furthermore, the mean percentage of the free target concentration above 2 mg·L^−1^ on the left side was 0.9 ± 0.2 for 0–10 h, indicating that, despite the higher dosing, the target concentrations were not maintained for the duration of the study in all subjects.

CPB may result in pharmacokinetic changes to cefazolin, for example, an increased volume of distribution [51,52]. Mechanisms include haemodilution, hypothermia, sequestration by the CPD circuit, and the induction of a systemic inflammatory response [53]. Deep hypothermic circulatory arrest was not explored in the included papers and may impact cefazolin pharmacokinetics [54]. Further studies with a focus on current dosing regimens, prolonged surgeries, and mammary artery harvesting are needed to describe tissue concentrations, providing clinicians with robust dosing recommendations for this population.

Most studies in the obstetric population undergoing caesarean sections [31,32,34,35,36,37,41], including those with obesity receiving a 2 g dose [31,32,34,36,37,41], reported a lower limit of variability tissue concentration > 2 mg·L^−1^ at incision and closure. Some studies did indicate that individual concentrations were not above 2 mg·L^−1^ for all patients with obesity [36,37] for the study period. Furthermore, MCS suggested that a repeat dose of cefazolin at 2 h is required for adequate target attainment in obese women undergoing caesarean section [37].

The results in the orthopaedic [41,42,43,44], abdominal surgery [10,45], and vascular surgery [46] populations were reassuring but sparse. Studies in the orthopaedic surgery group indicated that the lower limits of variability in tissue were >2 mg·L^−1^ at incision and closure following a 1 g [41] and 2 g [1,42,43] cefazolin bolus. Whilst tourniquet use did result in statistically different tissue concentrations [43] at the operative site, the lower limits of variability were still greater than target concentrations.

There are several limitations to this systematic review. We used the mean–SD, lower quartile, or lower range to infer adequate concentrations across a population. This primary outcome measure was chosen, as most studies reported a mean/median with a measure of variability as the primary outcome and did not provide the full range of data to enable alternative calculations. We acknowledge that if the lower limit of variability is above 2 mg·L^−1^ there still may be some patients with unbound plasma or tissue concentrations below this threshold at a higher risk for SSIs. Using other parameters, such as the entire distribution above the target concentration, would give more conservative estimates of target attainment for the population and could be considered in future studies. Furthermore, the application of Monte Carlo simulation (a statistical approach whereby a pharmacokinetic model can be used to simulate a larger population group) should be more consistently used to test the likely effectiveness of different dosing regimens. However, limited studies have applied this approach.

The optimal therapeutic pharmacokinetic/pharmacodynamic target to prevent SSIs is not well defined. The ideal target for an individual is the true MIC of the potential pathogen. In this review, we defined the target as an unbound cefazolin concentration > 2 mg·L^−1^, ECOFF value for *Staphylococcus aureus* [5]. The choice of ECOFF as a universal PK/PD target may also be too narrow, and achieving concentrations of 2 mg·L^−1^ will not prevent infections caused by organisms with higher MICs. Indeed, Gram-negative organisms with higher MICs are more relevant to particular surgery subtypes, such as abdominal and vascular surgery, and some authors have ascribed higher concentrations [14,15,25].

There is a heterogeneity between the included studies. The study design, populations, dosing regimens, and the lower limits of variability used (range vs. lower quartile vs. mean–SD) varied widely within surgical groups. Additionally, the tissue sampling technique is an important source of heterogeneity. Microdialysis measures the unbound ISF cefazolin concentration, whereas homogenised adipose tissue samples provide an average of the ISF, intracellular, and blood capillary cefazolin concentrations [7]. The limited number of RCTs, small number and sample size of the included studies, and methodological heterogeneity precluded meta-analysis. Outcomes were pharmacokinetic, with no correlation between concentrations and the actual SSI incidence. Consequently, it was difficult to make direct comparisons and draw conclusions about which dosing regimen is optimal.

## 4. Materials and Methods

### 4.1. Study Protocol/Registration and Reporting

This systematic review assessing the pharmacokinetics of cefazolin when administered for prophylaxis in elective surgery was conducted according to the PRISMA 2020 guidelines. Please see the Table S2 PRISMA 2020 abstract checklist and Table S3 PRISMA 2020 checklist in the Supplementary Materials [55]. The protocol was registered in PROSPERO (CRD42021080289). The protocol was amended to refine the PICOS criteria and to detail the risk of bias and quality assessment. This is documented in the version history.

### 4.2. Search Methodology/Search Strategy

A comprehensive search was performed on the 9 September 2024 for appropriate search terms in the title, abstracts, MeSH subject headings, author-supplied keywords, and controlled subject headings of scholarly publications in MEDLINE (PubMed). Search terms and search syntax in MEDLINE (PubMed) format are included in Table S4, Supplementary Materials. The search strategy was adapted further, including the inclusion of equivalent controlled subject terms, for Embase, CINAHL Complete, and CENTRAL (Table S5, Supplementary Materials). The strategy was adapted for the Clinical Knowledge Network EBSCO EDS discovery layer and run across multiple additional databases (Table S6, Supplementary Materials). Further Boolean and algorithmic searches were conducted with Google and Google Scholar, and hand searching of reference lists was utilised for the identification of grey literature. Searches were re-run before the final analysis in August 2025 to identify any further studies below.

### 4.3. Study Inclusion and Exclusion Criteria/Eligibility Criteria

All peer-reviewed, observational or randomised control trials were included if they met the following criteria:

- Intravenous cefazolin of any dose or regimen was administered for surgical site infection prophylaxis.
- Adult patients 18 years or older, with no upper age limit, were studied.
- Patients undergoing elective surgery only were studied.
- Cefazolin subcutaneous adipose tissue concentrations (using either homogenised adipose tissue samples or microdialysis techniques) and/or unbound plasma cefazolin concentrations at the time of skin incision and/or closure were measured.
- The mean or median concentrations and the range, IQR, or SD were reported.

Studies were excluded if they met the following criteria:

- They were in a language other than English.
- They included emergency surgery.
- Cefazolin was administered for any reason other than surgical site infection prophylaxis or if any patients received cefazolin in the 48 h prior to the prophylactic dose.

Studies were grouped by surgical subtype. If a study included more than one surgical subtype and the results of each surgery type were presented separately, the studies were included in both surgical subtype sections. If the results of different surgical subtypes were combined, the study was included under the surgery subtype section of the most common surgical subtype.

### 4.4. Primary Outcome

The primary outcome was the achievement of a lower limit of variability in unbound plasma and tissue cefazolin concentrations at the time of surgical incision and closure greater than the ECOFF value for *Staphylococcus aureus* [5], 2 mg·L^−1^, as determined by the European Committee on Antimicrobial Susceptibility Testing [5]. The lower limit of variability was defined as the first quartile, the mean minus the standard deviation, the lower range, or the mean minus the 95% confidence interval.

Studies that calculated unbound plasma concentrations based on assumed protein binding percentages were excluded. Where plasma and/or tissue sampling extended beyond the surgical duration, the closure concentration was considered as close as possible to the upper range, upper quartile, or upper standard deviation of the surgical duration. Where concentrations were presented in figures rather than exact numbers, we approximated the concentration based on the figures available.

### 4.5. Data Extraction and Quality Appraisal

Titles and/or abstracts of studies were retrieved using the search strategy, and those from additional sources were screened independently by two reviewers to identify studies that potentially met the inclusion criteria outlined above. The full text of these was retrieved and individually assessed for eligibility by two review team members. Discrepancies were resolved through the discussion between the two reviewers.

A standardised pre-piloted form was used to extract data from the included studies for the assessment of study quality. Extracted information included study population and characteristics (including author’s name, year of publication, country, study design, and sample size; eligibility criteria; surgical subtype and surgery; patient demographics and characteristics (including sex, age, weight, BMI, indices of renal function); cefazolin dose/infusion/redosing; recruitment and study completion rates; unbound plasma and/or adipose tissue concentrations and measurement intervals (number, confidence interval, interquartile range, *p* values); information for the assessment of risk of bias). There are no validated tools to assess the risk of bias in observational pharmacokinetic studies in the peri-operative period for prophylaxis antimicrobials; therefore, a formal risk of bias assessment tool was not used. A detailed narrative of the risk of bias assessment of individual studies was conducted and tabulated. Two reviewers extracted the data independently.

### 4.6. Data Synthesis

Studies were grouped according to surgical subtype and the data from selected studies were presented, including the study citation and year, study design, number of participants, patient characteristics, and mean/median concentrations and lower limit of variability, in tabular form with an accompanying narrative description.

A meta-analysis was not performed due to the small number and size of the studies, as well as heterogeneity between the studies.

An overall summary as to whether a 2 g cefazolin bolus achieves target unbound plasma and tissue concentration lower limits of variability was made for each surgical subgroup. The summary outcomes were defined as yes—almost all data supports that a concentration of >2 mg·L^−1^ is achieved; likely—most of the data supports that a concentration of >2 mg·L^−1^ is achieved; or insufficient data—there is insufficient evidence to support that a concentration of 2 mg·L^−1^ is achieved. This summary was presented as both a narrative in the results and in the Summary of Findings (SoF) table.

### 4.7. Certainty of Evidence

We used the Grading of Recommendations, Assessment, Development, and Evaluations (GRADE) approach to assess the certainty of evidence in each surgical subtype. This systematic review aimed to determine if target unbound plasma and tissue concentrations are obtained following prophylactic cefazolin administration. Observational studies measuring target concentrations were, therefore, considered the most appropriate study design [56], and observational studies were initially considered high-certainty evidence. Subsequently, the domains described in the GRADE handbook [57] were used to downgrade and upgrade certainty. An overview is presented in the SoF table.

## 5. Conclusions

Whilst it is reassuring that the lower limit of variability in tissue was greater than 2 mg·L^−1^ at incision and closure in most studies across the different surgical groups, there is evidence that some patients may not achieve target concentrations with the current dosing regimens, particularly in the cardiac surgery population. Furthermore, the necessity for increased dosing regimens in the bariatric surgery population group remains unclear.

The results in the obstetric population are reassuring; however, there is some evidence that not all patients are reaching target concentrations, and outcome studies are required. The number of patients included in abdominal and vascular surgery groups is low and should be further investigated.

## 6. Future Directions

Until large randomised controlled trial data become available, population pharmacokinetic modelling and dosing simulations may provide dosing guidance and predict the probabilities of peri-operative target attainment in different surgical populations.

## Figures and Tables

**Table 1:** Key findings of bariatric surgery studies.

Study Type	Participants (*n*)	Cefazolin Dosing Regimen	Mean/Median Patient Demographics Age (Years) TBW/BMI (kg/kg·m^−2^)	Mean/Median Surgical Duration (mins)	Lower Limit of Variability Unbound Plasma Concentration (Mean/Median) (mg·L^−1^)		Lower Limit of Variability Subcutaneous Tissue Concentration (Mean/Median) (mcg·g^−1^ or mg·L^−1^)			First Author, Date (Reference)
					Incision	Closure	Tissue Type	Incision	Closure	
Prospective, observational (no comparator group) (USA)	13	1 g 2 h before surgery, 1 g at induction	Age: 35.3 ± 8 TBW: 128.8 ± 20	148 ± 54	NA	NA	HA	3 (5.5 ± 2.5)	1.9 (3.5 ± 1.6)	Pories, 1981 [18]
Prospective, observational (no comparator) (Netherlands)	20	2 g at induction	Age: 44 ± 11 TBW: 151 ± 35 BMI: 51 ± 10	83 ± 24	At 30 min post-dose: >15 (approximately 35) ^@^	At 240 min post-dose: >2 (approximately 6) ^@^	NA	NA	NA	Van Kralingen, 2011 [13]
Prospective, observational (no comparator) (Brazil)	18	2 g 20.2 ± 8.6 min before incision then 1 g over 2 h	Age: 39.4 ± 11.6 BMI: 40.6 ± 4.0	95.8 ± 18.7	NA	NA	HA	4.1 (6.7 ± 2.6)	5.4 (7.9 ± 2.5)	Anlicoara, 2014 [20]
Prospective, observational (no comparator) (USA)	37	2 g over 3–5 min approximately 15 min before incision	Age: 45 ± 13 BMI: 46 ± 8 TBW: 127 ± 29	Time from dose to end of surgery	NA	NA	HA	All procedures: 3.7 (8.8 ± 5.0)		Chen, 2017 [21]
				LSG group: 108 ± 40					LSG group: 2.7 (6.9 ± 4.2)	
				LSG + other group: 108 ± 40					LSG + other group: 3.1 (6.1 ± 3.0)	
				RYGB group: 167 ± 45					RYGB group: 3.7 (8.1 ± 4.4)	
Prospective, observational (no comparator) (Australia)	12	2 g bolus 0–60 min before incision	Age: 41 ± 7.4 BMI: 50.0 ± 11.2 TBW: 148.0 ± 34.6	92.5 (70–175)	At 30 min post-dose: >20 (approximately 25) ^@^	At 300 min post-dose: >5 approximately 10) ^@^	MD	At 30 min post-dose: >2 (approximately 5) ^@^	At 180 min post-dose: approximately 3 (approximately 5) ^@^	Ryan, 2022 [17]
Prospective, comparative (weight comparator) (USA)	Group A: 17	2 g 30–60 min before incision, 2 g at 3 h post-dose	Group A: Age: 45.8 ± 9.5 TBW: 128.5 ± 14.3 BMI: 47 ± 1.3	Group A: 207 ± 34.8	NA	NA	HA	Group A: 1.9 (4.7 ± 2.8)	Group A: 2.5 (6.9 ± 4.4)	Edmiston, 2004 [12]
	Group B: 11		Group B: Age: 42.1 ± 13.2 TBW: 145.7 ± 19.1 BMI: 53.9 ± 2.8	Group B: 230.9 ± 43.5				Group B: 0.8 (3.2 ± 2.4)	Group B: 0.7 (4.0 ± 3.3)	
	Group C: 10		Group C: Age: 40.5 ± 8.4 TBW: 191.9 ± 50.3 BMI: 69.2 ± 10.2	Group C: 236.9 ± 44.2				Group C: 1.0 (2.6 ± 1.6)	Group C: 1.5 (3.6 ± 2.1)	
Prospective, comparative (weight comparator) (Netherlands)		2 g 15.6 ± 4.3 min before incision			At 30 min post-dose	At 120 min post-dose	MD	At 30 min post-dose	At 120 min post-dose	Brill, 2014 [11]
	Non-obese group: 7 ^#^		Non-obese group Age: 53.7 ±6.3 BMI: 28.2 ± 2.8 TBW: 86.2 ± 13	79.4 ± 14	Non-obese: approximately 30 (approximately 35) ^@^	Non-obese: >15 (approximately 17) ^@^		Non-obese group: approximately 20 (approximately 30) ^@^	Non-obese group: >5 (approximately 10) ^@^	
	Obese group: 8		Obese group Age 40.1 ± 55 BMI: 47.0 ± 5.8 TBW: 140.4 ± 23	74.1 ± 19	Obese: >20 (approximately 35) ^@^	Obese: approximately >5 (>5)		Obese group: approximately 20 (approximately 20) ^@^	Obese group: difficult to determine, likely > 2 (approximately 5) ^@^	
Prospective, comparative (weight comparator) (Belgium)		2 g over 30 min, 30–60 min before incision		Time from dose to end of surgery	At 30 min post-dose	At 240 min post-dose for all patients (*n* = 17): difficult to determine, likely greater than 2 (>4) ^@^	NA	NA	NA	Hites, 2016 [14]
	BMI < 35 group: 20 ^+^		BMI < 35 group: Age: 48 ± 14 BMI: 28 ± 5 TBW: 80 ± 15	BMI < 35 group: 180 (144–270)	BMI < 35 group: >20 (>50) ^@^					
	BMI > 35 group: 43		BMI > 35 group: Age: 44 ± 10 BMI: 43 ± 5 TBW: 122 ± 20	BMI > 35 group: 120 (105–135)	BMI > 35 group: >15 (>40) ^@^					
Prospective, comparative (weight comparator) (France)		4 g over approximately 9 min				At procedure end	HA			Cinotti, 2018 [15]
	Group A: 79	Group A: 39.7 ± 19.8 min before incision	Group A: Age: 42.7 ± 10.9 BMI: 44.0 ± 2.5 TBW: 124.3 ± 14.7	Group A: 72 ± 21.3	Group A: approximately >30 ^^^ (40) ^@^	Group A: Approximately 17 (20) ^@^		Group A: 6.8 (12.2 ± 5.4)	Group A: 4.1 (9.0 ± 4.9)	
	Group B: 37	Group B: 35.4 ± 14.8 min before incision	Group B: Age: 41.8 ± 10.5 BMI: 54.3 ±4.1 TBW: 143.2 ± 16	Group B: 79.6 ± 26.1	Group B: approximately >20 ^^^ (30) ^@^	Group B: Approximately >10 (12) ^@^		Group B: 5.9 (12.0 ± 6.1)	Group B: 3.6 (7.8 ± 4.2)	
Prospective, comparative (weight comparator) (Germany)		2 g over 25 min (median), starting 30 min (median) before incision			At 60 min post-dose	At 240 min post-dose	MD	At 30 min post-dose	At 270 min post-dose	Dorn, 2021 [10]
	Group A: 15		Group A: Age: 40.5 (25–65) ^a^ BMI: 51.7 (39.5–69.3) ^a^ TBW: 155 (123–200) ^a^	Group A: 162 (84–234) ^a^	Group A: >16 (approximately 20) ^@^	Group A: approximately 6 (approximately 8) ^@^		Group A: approximately 8 (>10) ^@^	Group A: approximately 4 (approximately 7) ^@^	
	Group B: 15 ^#^		Group B: Age: 45 (21–65) ^a^ BMI: 26.0 (18.7–29.8) ^a^ BW: 78 (50–96) ^a^	Group B: 162 (50–480) ^a^	Group B: > 25 (approximately 32) ^@^	Group B: approximately 6 (approximately 8) ^@^		Group B: >16 (approximately 30) ^@^	Group B: approximately 4 (approximately 8) ^@^	
Prospective, comparative (dose comparator) (Brazil)					At 10 min post-dose	At 240 min post-dose	MD	At 20 min post-dose	At 240 min post-dose	Palma, 2018 [16]
	2 g group: 4	2 g group: 2 g bolus 15 min before incision	2 g group: Age: 49.0 ± 5.7 BMI: 49.7 ± 5.4 TBW: 131.8 ± 24.7	2 g group, 199.3 ± 44.9	2 g group: >50 (>50) ^@^	2 g group: >8 (approximately 10) ^@^		2 g group: >8 (approximately 10) ^@^	2 g group: >7 (approximately 10) ^@^	
	3 g group: 5	3 g group: 3 g bolus 15 min before incision	3 g group: Age: 36.0 ± 11.8 BMI: 44.0 ± 5.1 TBW: 120.4± 20.0	3 g group, 199.0 ± 53.5	3 g group: >50 (>50) ^@^	3 g group: >10 (>10) ^@^		3 g group: > 10 (approximately 20) ^@^	3 g group: approximately 10 (>10) ^@^	
Prospective, comparative * (weight and dose comparator group) (Canada)	Group A: 8	Group A: 1 g	Group A: Age: 49 ± 14 TBW: 64.5 ± 9.6 BMI: 22 ± 4.	-	NA	NA	HA	Group A: 4.7 (6.0 ± 1.3)	Group A: 3.3 (4.1 ± 0.8)	Forse, 1989 [19]
	Group D: 11	Group D: 1 g	Group D: Age: 39 ± 6 TBW: 127.3 ± 18.6 BMI: 47 ± 6					Group D: 1.7 (4.0 ± 2.3)	Group D: 1.4 (2.4 ± 1.0)	
	Group E: 10	Group E: 2 g administered 12–15 min pre-incision for all groups	Group E: Age: 38 ± 7 TBW: 127.3 ± 16.8 BMI: 47 ± 5					Group E: 4.2 (7.3 ± 3.1)	Group E: 3.0 (4.1 ± 1.1)	

Except where otherwise indicated, data are means, means ± standard deviation, or median [interquartile range]. TBW, total body weight (kg); BMI, body mass index (kg·m^−2^); HA, homogenised adipose tissue; MD, microdialysis; LSG, laparoscopic sleeve gastrectomy; RYGB, Roux-en-Y gastric bypass. ^@^ Approximated from figure. ^#^ All patients were undergoing non-bariatric abdominal surgery (see Section 2.7 abdominal surgery). ^+^ Includes 12 patients undergoing non-bariatric abdominal surgery. ^ Lower end of 95% confidence interval. ^a^ median (min–max). * Patients receiving IM and S/C cefazolin are excluded from this analysis.

**Table 2:** Key findings from cardiothoracic surgery studies.

Study Type	Participants (*n*)	Cefazolin Dosing Regimen	Mean/Median Patient Demographics Age (Years) TBW/BMI (kg/kg·m^−2^)	Surgical Duration (mins)	CPB Time (mins)	Lower Limit of Variability Unbound Plasma Concentration (Mean/Median) (mg·L^−1^)		Lower Limit of Variability Subcutaneous Tissue Concentration (Mean/Median) (mcg·g^−1^ or mg·L^−1^)			First Author, Date (Reference)
						Incision	Closure	Tissue Type	Incision	Closure	
Prospective, observational (no comparator) (Austria)	7	4 g at least 60 min before skin incision, 2 g at skin closure	Age: 30 ± 13 TBW: 76 ± 12	188 ± 39	96 ± 32	NA	NA	MD	At 60 min post-dose: approximately 20 (approximately 60) ^@^	At 250 min post first dose: >20 (approximately 50) ^@^	Hutschala, 2007 [22]
Prospective, observational (renal function comparator) (Japan)	CrCl > 50: 35	2 g, 30 min within skin incision, then 1 g every 6 h Further 1 g in priming solution for CPB (*n* = 27)	CrCl > 50: Age: 60 ±10 BMI: 23 ± 4 TBW: 61 ± 12	CrCl > 50: 398 ± 95	NR	CrCl > 50: >20 (approximately 50) ^@^	CrCl > 50: difficult to determine, likely <2 (approximately 10) ^@^	NA	NA	NA	Kosaka, 2012 [23]
	CrCl = 10–49: 19		CrCl = 10–49: Age: 71 ± 11 BMI: 24 ± 5 TBW: 60 ± 16	CrCl = 10–49: 393 ± 95		CrCl = 10–49: >30 (approximately 60) ^@^	CrCl = 10–49: >5 (approximately 30) ^@^				
	Dialysis: 8		Dialysis: Age: 69 ± 7 BMI: 20 ± 2 TBW: 53 ± 7	Dialysis: 444 ± 112		Dialysis: > 50 (approximately 80) ^@^	Dialysis: > 20 (approximately 60) ^@^				
Prospective, observational (no comparator) (Austria)	8	4 g 1 h before skin incision and 2 g approximately 60 min before skin closure	Age: 69 ± 11 BMI: 27.2 ± 2.7	NR	NR	NA	NA	HA		At 600 min	Andreas, 2013 [24]
									Right sternal: > 15 (approximately 20) ^@^	Right sternal: approximately 5 (>8) ^@^	
									Left sternal: 7.3 (13.1 ± 5.8) ^@^	Left sternal: approximately 0.2 (>4) ^@^	
Prospective, observational (no comparator) (USA)	10	2 g within 1 h of incision, then 2 g every 3 h; 1 g into CPB circuit	Age: 62 ± 6 TBW: 85 ± 28	241 ± 47	116 ± 40	31 (65 ± 34)	23 (59 ± 36)	NA	NA	NA	Hollis, 2015 [25]
Prospective, observational (no comparator) (Brazil)	19	2 g at induction, 1 g every 4 h	Age: 60.3 y BMI: 80% of patients had BMI < 30, 15% of patients had BMI 30–40, 5% of patients had BMI > 40%	NR	NR	NA	NA	HA	3.9 (6.1 ± 2.2)	5.5 (8.4 ± 2.9)	Tchaick, 2017 [26]
Prospective, observational (no comparator) (Japan)	27	1 g before skin incision, then 1 g every 4 h; 2 g at start of CPB	Age: 70 ± 12 –TBW: 62 ± 12	428 ± 113	206 ± 51	11 (17 (11–35)) ^	21 (70 (21–137)) ^	NA	NA	NA	Asada, 2018 [27]
Prospective, observational (no comparator) (Canada)	40	Mean dose: 23.5 ± 5.4 mg·kg^−1^, 35 ± 13 min before incision	Age: 65 ± 10 TBW: 88 ± 16	278 ± 74	NR	NA	6.6 (32.8 ± 26.2)	NA	NA	NA	Zelenitsky, 2018 [28]
Prospective, observational (compared to Andreas, 2013 [24]) (Austria)	8	4 g, over 30 min given 3 h and 1 h before skin incision, 2 g during skin closure	Age: 69 ± 7 BMI: 26.3 ± 3.9	NR	NR	NA	NA	MD	Right side: 8.1 (12.8 ± 4.1) Left side: 8.6 (11.2 ± 2.6)	At 600 min: Left side: approximately 1 (>2)	Andreas, 2020 [29]
Prospective, observational (no comparator) (Canada)	16	2 g 30 min before skin incision, a further 2 g at 4 h	Age: 44 ± 12 TBW: 75 ± 16 BMI: 28 ± 7		165 ± 52	>50 (>60) ^@^	At 210 min >20 (approximately 30) ^@^	NA	NA	NA	Alli, 2023 [30]

Except where otherwise indicated, data are means, means ± standard deviation, or median [interquartile range]. Age (years); TBW, total body weight (kg); BMI, body mass index (kg·m^−2^); CPB, cardiopulmonary bypass; HA, homogenised adipose tissue; MD, microdialysis; NA, not applicable; NR, not reported; CrCl, cre-atinine clearance (mL·min^−1^). ^@^ Approximated from the figure. ^ Median (range).

**Table 3:** Key findings from obstetric surgery studies.

Study Type	Participants (*n*)	Cefazolin Dosing Regimen	Mean/Median Patient Demographics Age (Years) TBW/BMI (kg/kg·m^−2^)	Surgical Duration (mins)	Lower Limit of Variability Unbound Plasma Concentration (Mean/Median) (mg·L^−1^)		Lower Limit of Variability Subcutaneous Tissue Concentration (Mean/Median) (mcg·g^−1^ or mg·L^−1^)			First Author, Date (Reference)
					Incision	Closure	Tissue Type	Incision	Closure	
RCT (New Zealand)	2 g group: 11	2 g group: 2 g at “pre-operative checklist”	2 g group: Age: 31.1 ± 6.5 TBW: 117.8 ±23 BMI: 46.0 ± 6.0	2 g group: 68.1 ± 13.5	NA	NA	HA	2 g group: 11.7 (18.4 ± 6.7)	2 g group: 15.7 (31.7 ± 16.0)	Stitely, 2013 [31]
	4 g group: 9	4 g group: 4 g at “pre-operative checklist”	4 g group: Age: 30.3 ± 4.6 TBW: 107.8 ± 28.3 BMI: 43.2 ± 8.4	4 g group: 56.2 ± 14.8				4 g group: 16 (40.1 ± 24.1)	4 g group: 17.5 (34.9 ± 17.4)	
RCT (USA)	2 g group: 28	2 g group: 2 g 22 (19–28) min before incision	2 g group: Age: 30 (25.5–34.0) BMI: 38.9 (35.4–45.6)	2 g group: 62.5 (56.5–77.5)	NA	NA	HA	2 g group: 5.1 (9.4 (5.1–13.4))	2 g group: 4.4 (8.4 (4.4–13.2))	Maggio, 2015 [3]
	3 g group: 30	3 g group: 3 g 23 (20–28) min before incision	3 g group: Age: 32 (22–44) BMI: 39.3 (36.7–44.8)	3 g group: 65.0 (57.5–72.5)				3 g group: 7.0 (11.7 (7.0–18.3))	3 g group: 6.7 (8.7 (6.7–18.8))	
RCT (USA)	2 g group: 13	2 g group: 2 g 4 (2–6) min before incision	2 g group: Age: 29.0 (23.5–34.0) BMI: 42.9 (39.1–46.2)	2 g group: 59.8 (50.0–65.0)	NA	NA	HA	2 g group: 5.4 (7.4 (5.4–9.4))	2 g group: 9.2 (11.8 (9.2–16.2))	Young, 2015 [33]
	3 g group: 13	3 g group: 3 g 3 (1–10) min before incision	3 g group: Age 31.0 (30.0–35.0) BMI: 41.8 (37.3–44.6)	3 g group: 67.4 (56.0–72.0)				3 g group: 5.4 (12.0 (5.4–16.0))	3 g group: 10.8 (14.6 (10.8–20.9))	
Prospective, observational (weight comparator) (USA)	BMI < 30 group: 10	2 g 30–60 min before incision	BMI < 30 group: Age: 28.3 ± 5.6 Weight: 65.7 ± 4.8 BMI: 26.7 ± 1.3	BMI < 30 group: 60.9 ± 14.1	NA	NA	HA	BMI < 30 group: 6.7 (9.4 ± 2.7)	BMI < 30 group: 2.7 (9.1 ± 6.4)	Pevzner, 2011 [34]
	BMI 30–39.9 group: 10		BMI 30–39.9 group: Age: 32.8 ±7.1 TBW: 90.0 ± 10.8 BMI: 34.1 ± 2.6	BMI 30–39.9 group: 56.8 ± 21.5				BMI 30–39.9 group: 4.1 (6.4 ± 2.3)	BMI 30–39.9 group: 3.1 (6.6 ± 3.5)	
	BMI > 40 group: 9		BMI > 40 group: Age: 28.1 ± 4.6 TBW: 121.8 ± 16.1 BMI: 44.5 ± 4.5	BMI > 40 group: 56.2 ± 11.6				BMI > 40 group: 3.2 (4.4 ± 1.2)	BMI > 40 group: 3.2 (4.7 ± 1.5)	
Prospective, observational (weight comparator) (USA)		3 g 30–60 min before incision	Both groups: Age: 31.5 (26.5–36.5) Weight: 105.6 (87.2–120.0)	Both groups: 33.5 (25.0–53.0)	NA	NA	HA			Swank, 2015 [35]
	BMI 30–39.9 group: 14		BMI 30–39.9 group: BMI: 33.8 ± 2.9					BMI 30–39.9 group: 20.3 (22.4 (20.3–34.4))	BMI 30–39.9 group: 18.9 (24.8 (18.9–34.1))	
	BMI > 40 group: 14		BMI > 40 group: BMI: 45.0 ± 3.8					BMI > 40 group: 7.6 (9.6 (7.6–15.8))	BMI > 40 group: 6.2 (7.1 (6.2–9.8))	
Prospective, observational (dose and weight comparator) (USA)	2 g dosage group: 65	2 g dosage group: 2 g 30–60 min before skin incision	2 g dosage group: Age: 29.6 (28.1–31.1) BMI: 38.3 (37.3–39.3)	2 g dosage group: 77.1 (72.1–82.1)	NA	NA	HA	2 g dosage group: 3.0 (5.3 (3.0–9.6))	2 g dosage group: 2.8 (4.7 (2.8–7.6))	Kram, 2017 [36]
	3 g dosage group: 19	3 g dosage group: 30–60 min before skin incision	3 g dosage group: Age: 29.9 (26.6–31.2) BMI: 48.4 (46.5–50.4)	3 g dosage group: 75.2 (66.7–83.7)				3 g dosage group: 3.9 (6.4 (3.9–8.4))	3 g dosage group: 2.6 (6.9 (2.6–10.6))	
Prospective, observational (compared low and high QBL ^#^) (USA)	Low QBL group: 15	2 g if <100 kg; 3 g if > 100 kg within 60 min of skin incision	Low QBL group: Age 32.6 (27.3–35.1) BMI: 31.9 (25.6–35.2)	Low QBL group: 48 (37–59)	NA	NA	HA	Low QBL group: 4.1 (7.2 (4.1–11.0))	Low QBL group: 1.9 (3.5 (1.9–5.0)	Dotters-Katz, 2019 [38]
	High QBL group: 5		High QBL group: Age 32.9 (32.8–34.1) BMI: 38.1 (34.5–50.3)	High QBL group: 60 (52–65)				High QBL group: 3.6 (4.5 (3.6–9.7))	High QBL group: 3.0 (3.9 (3.0–5.5))	
Prospective, observational (no comparator) (Australia)	12	2 g within 30 min of skin incision	Age: 32.8 ± 4.8 TBW: 119.1 ± 18.8 BMI: 41.5 (39.7–46.6)	75.8 (21.0) Upper range: 125 min	At 30 min post-dose: >20 (approximately 25) ^@^	At 180 min post-dose: >2 (approximately 10) ^@^	MD	At 30 min post-dose: Difficult to determine, likely > 2 (approximately 10) ^@^	At 120 min post-dose: Difficult to determine, likely > 2 (approximately 5) ^@^	Eley, 2020 [37]

Except where otherwise indicated, data are means, means ± standard deviation, or median [interquartile range]. TBW, total body weight at time of delivery (kg); BMI, body mass index at time of delivery (kg·m^−2^); HA, homogenised adipose tissue; MD, microdialysis; NA, not applicable; QBL, quantitative blood loss. ^#^ Low QBL defined as QBL < 75th percentile of study population. High QBL defined as QBL > 75th percentile of study population. ^@^ Approximated from the figure.

**Table 4:** Key findings from orthopaedic and spinal surgery studies.

Study Type	Participants (*n*)	Cefazolin Dosing Regimen	Mean/Median Patient Demographics Age (Years) TBW/BMI (kg/kg·m^−2^)	Surgical Duration (mins)	Lower Limit of Variability Unbound Plasma Concentration (Mean/Median) (mg·L^−1^)		Lower Limit of Variability Subcutaneous Tissue Concentration (Mean/Median) (mcg·g^−1^ or mg·L^−1^)			First Author, Date (Reference)
					Incision	Closure	Tissue Type	Incision	Closure	
RCT (comparing tourniquet vs. no tourniquet) (Canada)		2 g within 5–60 min of incision			NA	NA	HA		At 90 min	Montreuil, 2024 [43]
	Tourniquet group: 29		Tourniquet group: Age: 69.3 ± 9.6 BMI: 29.3 ± 5.4	Tourniquet group: 77.8 ± 16.7				Tourniquet group: 5.0 (6.1 (5.0–7.2)) ^a^	Tourniquet group: 6.2 (8.4 (6.2–10.4)) ^a^ *p* = 0.03	
	Non-tourniquet group: 30		Non-tourniquet group: Age: 69.9 ± 9.7 BMI: 30.1 ± 5.5	Non-tourniquet group: 81.4 ± 19.0				Non-tourniquet group: 6.3 (10.5 (6.3–14.7)) ^a^	Non-tourniquet group: 7.0 (10.1 (7.0–13.2)) ^a^ *p*= 0.33	
Prospective, observational (no comparator) (Canada)	10	2 g at least 10 min before skin incision or tourniquet inflation 3 g if > 120 kg	NA	NA	NA	NA	HA	4.0 (7.4 ± 3.4)	NA	Russo, 2023 [44]
Prospective, comparator (comparing systemic vs. intraosseous route of administration) (People’s Republic of China)	30 ^#^	2 g 30 min before tourniquet inflation	Age: 68.9 ± 6.9 BMI: 25.6 ± 2.7		NA	NA	HA	9.5 (11.4 ± 1.9)	NA	Zhang, 2024 [42]
RCT * (USA)				NR	30 min post-dose ^@^	NR	NA	NA	NA	Naik, 2017 [39]
	Intermittent bolus: 10	Intermittent bolus: 2 g 15–60 min before incision, 2 g at 4 h	Intermittent bolus: Age: 62 (40–78) TBW: 80 ± 15 BMI: 27 ± 5		Intermittent bolus: >15 (approximately 30)					
	Continuous infusion: 10	Continuous infusion: 2 g 15–60 min before incision, 500 mg·h^−1^ infusion	Continuous infusion: Age: 54 (18–79) TBW: 82 ± 11, BMI: 27 ± 4		Continuous infusion: >30 (approximately 50) ^@^					
Prospective, comparator (comparing systemic vs. intraosseous route of administration) (USA)	11 ^#^	1 g 10–30 min before tourniquet inflation	Age: 65.3 (48–83) ^ BMI: 29.1 (23.1–35.0) ^	76 (39–110) ^	NA	NA	HA	2.9 (7.2 ± 4.3)	5.1 (11.3 ± 6.2)	Young, 2013 [41]
Prospective, observational (USA)	46 ^b^	1 g before surgery ^+^	Age: 54 ± 15 TBW: 87.8 ± 26.5 BMI: 29.8 ± 7.5	122 ± 120	NA	2.8 (8.1 ± 5.3)	NA	NA	NA	Koopman, 2007 [40]

Except where otherwise indicated, data are means, means ± standard deviation, or median (interquartile range). TBW, total body weight (kg); BMI, body mass index (kg·m^−2^); HA, homogenised adipose tissue; NA, not applicable. NR, not reported; ^a^ mean (95% confidence interval). ^#^ Participants who received regional cefazolin were excluded from this analysis. * Also included patients undergoing urological surgery. ^@^ Approximated from the figure. ^ Means (range). ^+^ One patient received 2 g cefazolin and one patient received 2 × 1 g doses before surgery. One repeat 1 g dose was given to four patients, and one patient received an extra two doses of 1 g during surgery. ^b^ Included 14 patients undergoing abdominal surgery.

**Table 5:** Key findings from urology, neurosurgery, vascular, and abdominal surgery studies.

Surgical Subtype	Study Type	Participants (*n*)	Cefazolin Dosing Regimen	Mean/Median Patient Demographics Age (Years) TBW/BMI (kg/kg·m^−2^)	Surgical Duration (mins)	Lower Limit of Variability Unbound Plasma Concentration (Mean/Median) (mg·L^−1^)		Lower Limit of Variability Subcutaneous Tissue Concentration (Mean/Median) (mcg·g^−1^ or mg·L^−1^)			First Author, Date (Reference)
						Incision	Closure	Tissue Type	Incision	Closure	
Abdominal surgery	Prospective, comparative (weight comparator) (Netherlands)		2 g 15.6 ± 4.3 min before incision			At 30 min post-dose	At 120 min post-dose	MD	At 30 min post-dose	At 120 min post-dose	Brill, 2014 [11]
		Non-obese group: 7		Non-obese group: Age: 53.7 ±6.3 BMI: 28.2 ± 2.8, TBW: 86.2 ± 13	79.4 ± 14	Non-obese: approximately 30 (approximately 35) ^@^	Non-obese: >15 (approximately 17) ^@^		Non-obese group: approximately 20 (approximately 30) ^@^	Non-obese group: >5 (approximately 10) ^@^	
		Morbidly obese group: 8 ^&^		Obese group: Age 40.1 ± 55, BMI: 47.0 ± 5.8, TBW: 140.4 ± 23	74.1 ± 19	Obese: >20 (approximately 35) ^@^	Obese: Approximately 10 (>5) ^@^		Obese group: approximately 20 (approximately 20) ^@^	Obese group: difficult to determine, likely >2 (approximately 5) ^@^	
Abdominal or pelvic surgery	Prospective, comparative (weight comparator) (Germany)		2 g over 25 min (median), starting 30 min (median) before incision			At 60 min post-dose	At 240 min post-dose	MD	At 30 min post-dose	At 270 min post-dose	Dorn, 2021 [10]
		Group A: 15 ^&^		Group A: Age: 40.5 (25–65) ^a^, BMI: 51.7 (39.5–69.3) ^a^ TBW: 155 (123–200) ^a^	Group A: 162 (84–234) ^a^	Group A: >16 (approximately 20) ^@^	Group A: approximately 6 (approximately 8) ^@^		Group A: approximately 8 (>10) ^@^	Group A: approximately 4 (approximately 7) ^@^	
		Group B: 15		Group B: Age: 45 (21–65) ^a^ BMI: 26.0 (18.7–29.8) ^a^ TBW: 78 (50–96) ^a^	Group B: 162 (50–480) ^a^	Group B: >25 (approximately 32) ^@^	Group B: approximately 6 (approximately 8) ^@^		Group B: >16 (approximately 30) ^@^	Group B: approximately 4 (approximately 8 ^@^	
Gastric surgery	Prospective, observational (no comparator) (South Korea)	40	2 g over 10 min before skin incision	Age: 58 ± 11 TBW: 67 ± 13	142 (42–240)	At 45 min: all samples were >5 ^#^	At 2 h: all samples were >2 ^#^	NA	NA	NA	Kim, 2022 [45]
Vascular surgery	Prospective, observational (no comparator) (Australia)	12	2 g 30 min before incision	Age: 70 (66–76) TBW: 88 (81–95)	180 (156–192)	At 30 min: >10 (approximately 20) ^@^	At 200 min: approximately 8 (approximately 10) ^@^	MD	At 200 min: >2 (approximately 5) ^@^	At 200 min: >10 (approximately 10) ^@^	Douglas, 2011 [46]

Except where otherwise indicated, data are means, means ± standard deviation, or median [interquartile range]. TBW, total body weight (kg); BMI, body mass index (kg·m^−2^); MD, microdialysis; NA, not applicable. ^&^ Bariatric surgery patients (also see Section 2.1 Bariatric surgery). ^@^ Approximation from the figure. ^#^ No mean/median given. ^a^ Median (min–max).

**Table 6:** Summary of Findings table.

Surgical Subgroup	Number of Participants (Studies)	Did 2 g Cefazolin Achieve a Lower Limit of Variability of >2 mg·L^−1^?	Certainty of Evidence (GRADE)	Comments
Bariatric surgery	378 (12)	Plasma: yes	●●●●	
		Tissue: likely	●●○○	Downgraded for risk of bias and inconsistency.
Cardiac surgery	197 (9)	Plasma: yes	●●●●	Downgraded for risk of bias due to limited studies using current dosing regimens; upgraded for large effect size.
		Tissue: insufficient evidence	●○○○	Downgraded for risk of bias, inconsistency, and external validity due to prolonged sampling time.
Caesarean delivery	277 (8)	Plasma: yes	●●●●	Downgraded for risk of bias due to limited studies; upgraded for large effect size.
		Tissue: yes	●●●●	
Orthopaedic and spinal surgery	176 (6)	Plasma: insufficient evidence	●○○○	Downgraded for risk of bias from limited number of studies and reduced external validity due to inclusion of non-orthopaedic surgical patients.
		Tissue: yes	●●●○	Downgraded for risk of bias from limited studies.
Abdominal surgery	62 (3)	Plasma: yes	●●●●	Downgraded for risk of bias; upgraded for large effect size.
		Tissue: insufficient evidence	●●○○	Downgraded for risk of bias due to limited number of studies.
Vascular surgery	12 (1)	Plasma: yes	●●○○	Downgraded for risk of bias due to limited number of studies.
		Tissue: yes	●●○○	Downgraded for risk of bias due to limited number of studies.

GRADE Working Group grades of evidence. ●●●● High certainty: There is a high confidence that the concentration lower limit of variability is >2 mg·L^−1^; ●●●○ Moderate certainty: There is moderate confidence that the concentration lower limit of variability is >2 mg·L^−1^; ●●○○ Low certainty: There is low confidence that the concentration lower limit of variability is >2 mg·L^−1^; ●○○○ Very low certainty: There is very low certainty that the concentration lower limit of variability is >2 mg·L^−1^.

## Data Availability

The raw data supporting the conclusions of this article will be made available by the authors on request.

## Supplementary Materials

The following supporting information can be downloaded at https://www.mdpi.com/article/10.3390/antibiotics14121227/s1. Table S1. Risk of bias assessment. Table S2. PRISMA 2020 abstract checklist. Table S3. PRISMA 2020 checklist. Table S4. Database search strategy MEDLINE(PubMed). Table S5. Database search strategy EMBASE. Table S6. CKN EBSCO EDS discovery layer additional databases.

## Abbreviations

The following abbreviations are used in the manuscript:

SSI	Surgical site infections
MIC	Minimum inhibitory concentration
ECOFF	Epidemiological cut-off
ISF	Interstitial fluid
TBW	Total body weight
BMI	Body mass index
CPB	Cardiopulmonary bypass
AUC	Area under the curve
PTA	Probability of target attainment
SD	Standard deviation
CD	Caesarean delivery
RCTs	Randomised controlled trials
SoF	Summary of Findings
