# Supplementary material for: Peri-Operative Dosage and Therapeutic Concentrations of Cefazolin Administered for Surgical Site Infection Prophylaxis in Elective Surgery—A Systematic Review
Source: Antibiotics (Basel). 2025 Dec 5;14(12):1227. doi: 10.3390/antibiotics14121227 (PMC12730041; doi:10.3390/antibiotics14121227)
Supplement: Supplementary file 1 [file antibiotics-14-01227-s001.zip › antibiotics-3992764-supplementary.pdf]

Supplementary Material

Table S1. Risk of bias assessment

**Surgical Subtype**	**First author, date (reference)**	**Risk of Bias Summary**
Bariatric Surgery	Pories, 1981 [18]	The age of the study may have bias' that affect external validity and applicability to today's bariatric surgical population ie these would have been open surgeries where as most procedures are currently laparoscopic and also older measurement technique.<br>Reduced dose compared to current recommendations- confounding bias. Measurement bias from homogenised adipose tissue sampling.
	Van Kralingen, 2011 [13]	Low risk of bias: n= 20, good total body weight/body mass index spread, Single 2 g cefazolin dose.
	Anlicoara, 2014 [20]	The results are confounded by a 2 g plus 1 g/h infusion and likely measurement bias from homogenised adipose tissue sampling. Otherwise low risk of bias.
	Chen, 2017 [21]	Measurement bias from homogenised adipose tissue sampling. Otherwise, low risk of bias: n= 37, good total body weight/body mass index spread, Single 2 g cefazolin dose.
	Ryan, 2022 [17]	Wide standard deviation indicating imprecise measurement, particularly ISF at 2 h. The standard deviation is lower at 2 h than 4 h without redosing raising question of measurement bias. Otherwise good total body weight/body mass index spread and 2 g dose given.
	Edmiston, 2004 [12]	Measurement bias from homogenised adipose tissue sampling. Long surgical duration compared to other studies may have confounded results.
	Brill, 2014 [11]	Only 7 patients in obese group and 8 patients in non-obese group, otherwise low risk of bias. Tissue measured using microdialysis and 2 g dose given.
	Hites, 2016 [14]	Low risk of bias: n = 63 good body mase index spread although in the BMI > 35 group the BMI mead +/- SD was 43 +/- 5 kg/m^2 so therefore may not include patients with a very large BMI > 50 kg/m^2
	Cinotti, 2018 [15]	Measurement bias introduced with homogenised tissue sampling. 4 g dose given which confounds the results. May not include many patients with BMI > 50 kg.m^2 Mean +/- SD BMI in Group A: 44 +/- 2.5 kg/m^2. Otherwise large study n = 117.
	Dorn, 2021 [10]	Low risk of bias: large study (n= 30), 2 g dose given, tissue measured via microdialysis.
	Palma, 2018 [16]	Small study (n=9) but otherwise low risk of bias: 2 and 3 g cefazolin administered, good body mass index spread.
	Forse, 1989 [19]	Older study and therefore may have bias that effects external validity and applicability to todays bariatric surgical population. N= 30 but only 10 patients received 2 g dose.

Cardiac Surgery	Hutschala, 2007 [22]	Small study size (n=7), increasing the risk of bias. 4 g dose administered confounding results and external validity given current dosing recommendations.
	Kosaka, 2012 [23]	This is a large study (n= 62) which reduces the potential for systemic bias. The authors examined the impact of renal impairment which is an important confounder in cefazoin pharmacokinetics and a relevant comorbidity in the cardiac surgical population, improving external validity. This study doesn't report cardiopulmonary bypass time, an important potential confounder on cefazolin pharmacokinetics.
	Andreas, 2013 [24]	Small study (n=8). Tissue concentrations were measured via homogenised tissue sampling introducing measurement bias. Duration of surgery not recorded and therefore reporting bias reduces ability to apply appropriate time of closure sampling in this systematic review. Examines the confounding factor of location of tissue sampling, particularly in the context of left mammary artery harvesting. This study doesn't report cardiopulmonary bypass time, an important potential confounder on cefazolin pharmacokinetics.
	Hollis, 2015 [25]	Low risk of bias (n=10) 2 g given at incision and then 2 g every 3h. Cardiopulmonary bypass time reported.
	Tchaick, 2017 [26]	Measurment bias introduced by homogenised adipose tissue sampling Otherwise low risk of bias: n=11, 2 g cefazlin at induction and further 1 g at 4 h. This study doesn't report cardiopulmonary bypass time, an important potential confounder in cefazolin pharmacokinetics.
	Asada, 2018 [27]	Dose administered was 1 g at induction and then 1 g every 4 h with additional 2 g at start of bypass, therefore confounding results when compared with current recommendations. This study doesn't report cardiopulmonary bypass time, an important potential confounder in cefazolin pharmacokinetics.
	Zelenitsky, 2018 [28]	Large sample size (n=40). Only unbound plasma concentration at closure lower limit of variability stated.
	Andreas, 2020 [29]	Small study (n=8) increasing risk of systemic bias. Duration of surgery not recorded and therefore reporting bias reduces ability to apply appropriate time of closure sampling in this systematic review. Examines the confounding factor of location of tissue sampling, particularly in the context of left mammary artery harvesting. This study doesn't report cardiopulmonary bypass time, an important potential confounder on cefazolin pharmacokinetics.
	Alli, 2023 [30]	Uses current dosing recommendations. Low risk of bias: n= 16, current cefazolin dosing used. Cardiopulmonary bypass time recorded.
Cesarean Delivery	Stitely, 2013 [31]	RCT. Patients were randomised to 2 g or 4 g of cefazolin. Randomisation was not blinded but there is a low risk of bias as observational study measuring tissue concentrations. 2 g group had some patients with TBW > 120 and therefore would confound results. Measurement bias as tissue concentrations measured with homogenised adipose tissue sampling.
	Maggio, 2015 [32]	Low risk of bias. Blinded RCT. Patients were randomised to 2 g or 3 g of cefazolin. 1 patient excluded due to protocol violation. Therefore, didn't follow intention to treat. Large study (n=58). Measurement bias introduced with homogenised adipose tissue sampling.

	Young, 2015 [33]	Low risk of bias. Double-blinded RCT. Obese patients were randomised to receive 2 or 3 g. Measurement bias with homogenised adipose tissue sampling.
	Pevzner, 2011 [34]	Measurement bias from homogenised adipose tissue sampling. Otherwise low risk of bias (n= 29), 2 g cefazolin administered.
	Swank, 2015 [35]	Measurement bias with homogenised adipose tissue sampling. Adequate sample size (n=28) to reduce risk of bias. 3 g dose administered to both patient groups. Lower BMI group would have patietns less than 120 kg and therefore may confound results and affect external validity.
	Kram, 2017 [36]	Measurement bias with homogenised adipose tissue sampling. Otherwise a low risk of bias: patients were dosed according to current guidelines and a large sample size (n=84)
	Dotters-Katz, 2019 [38]	Patients administered 2 g if < 100kg and 3 g if > 100 kg total body weight. Measurement bias with homogenised adipose tissue sampling. Samples were analysed according to blood loss. The low blood loss group had lower quartile range lower than the high blood loss group but this is likely due to variability across the population and inadequate power.
	Eley, 2020 [37]	Only caesarean delivery study that used microdalysis for tissue concentration measurement. Good sample size (n=12). Only studies the obese patients and used a 2 g dose.
Orthopedic and Spinal Surgery	Montreuil, 2024 [43]	Unblinded RCT comparing tissue concentrations in tourniquet vs no tourniquet. Measurement bias with homogenised adipose tissue sampling. Large sample size (n=59)
	Russo, 2023 [44]	Measurement bias with homogenised adipose tissue sampling. Adequate sample size (n=10) using current dosing recommendations. Only tissue concentrations at incision measured.
	Zhang, 2024 [42]	Measurement bias with homogenised adipose tissue sampling. Large sample size (n=30) using current dosing recommendations. Only tissue concentrations at incision measured.
	Naik, 2017 [39]	2 g before incision and then 2 g at 4 h was compared to 2 g at incision and 500 mg/h infusion. Included patients undergoing urological surgery introducing bias and reducing external validity to patients undergoing spinal surgery. Sample size adequate (n=20)
	Young, 2013 [41]	Measurement bias with homogenised adipose tissue sampling. Adequate sample size (n=10) only 1 g dose was given confounding the results.
Abdominal Surgery	Koopman, 2007 [40]	Included 14 patients undergoing abdominal surgery confounding results and reducing applicability to the orthopedic surgical population. Only 1 g cefazolin was administered.
	Kim, 2022 [45]	Low risk of bias: adequate sample size (n=20), Unbound plasma samples reported only.
Vascular Surgery	Douglas, 2011 [46].	Selection bias: only included patients undergoing elective abdominal aortic aneurysm repair were included and therefore results may to extend to patients undergoing different types of vascular surgery. Otherwise, low risk of bias: adequate sample size (n=12), tissue concentrations taken by microdialysis.

Table S2. PRISMA 2020 abstract checklist

Section and Topic	Item #	Checklist item	Reported (Yes/No)
**TITLE**			yes
Title	1	Identify the report as a systematic review.	
**BACKGROUND**			
Objectives	2	Provide an explicit statement of the main objective(s) or question(s) the review addresses.	yes
**METHODS**			
Eligibility criteria	3	Specify the inclusion and exclusion criteria for the review.	Yes
Information sources	4	Specify the information sources (e.g. databases, registers) used to identify studies and the date when each was last searched.	Yes
Risk of bias	5	Specify the methods used to assess risk of bias in the included studies.	Yes
Synthesis of results	6	Specify the methods used to present and synthesise results.	Yes
**RESULTS**			
Included studies	7	Give the total number of included studies and participants and summarise relevant characteristics of studies.	yes
Synthesis of results	8	Present results for main outcomes, preferably indicating the number of included studies and participants for each. If meta-analysis was done, report the summary estimate and confidence/credible interval. If comparing groups, indicate the direction of the effect (i.e. which group is favoured).	yes
**DISCUSSION**			
Limitations of evidence	9	Provide a brief summary of the limitations of the evidence included in the review (e.g. study risk of bias, inconsistency and imprecision).	yes
Interpretation	10	Provide a general interpretation of the results and important implications.	yes
**OTHER**			
Funding	11	Specify the primary source of funding for the review.	yes
Registration	12	Provide the register name and registration number.	yes

Table S3 PRISMA 2020 checklist

Section and Topic	Item #	Checklist item	Location where item is reported
**TITLE**			
Title	1	Identify the report as a systematic review.	Line 2-4
**ABSTRACT**			
Abstract	2	See the PRISMA 2020 for Abstracts checklist.	Line 29-64
**INTRODUCTION**			
Rationale	3	Describe the rationale for the review in the context of existing knowledge.	Line 72-131
Objectives	4	Provide an explicit statement of the objective(s) or question(s) the review addresses.	Line 133-140
**METHODS**			
Eligibility criteria	5	Specify the inclusion and exclusion criteria for the review and how studies were grouped for the syntheses.	Line 797-819
Information sources	6	Specify all databases, registers, websites, organisations, reference lists and other sources searched or consulted to identify studies. Specify the date when each source was last searched or consulted.	Line 781-793
Search strategy	7	Present the full search strategies for all databases, registers and websites, including any filters and limits used.	Tables S3-S6
Selection process	8	Specify the methods used to decide whether a study met the inclusion criteria of the review, including how many reviewers screened each record and each report retrieved, whether they worked independently, and if applicable, details of automation tools used in the process.	Lines 847-851
Data collection process	9	Specify the methods used to collect data from reports, including how many reviewers collected data from each report, whether they worked independently, any processes for obtaining or confirming data from study investigators, and if applicable, details of automation tools used in the process.	Lines 853-864
Data items	10a	List and define all outcomes for which data were sought. Specify whether all results that were compatible with each outcome domain in each study were sought (e.g. for all measures, time points, analyses), and if not, the methods used to decide which results to collect.	Lines 832-844
	10b	List and define all other variables for which data were sought (e.g. participant and intervention characteristics, funding sources). Describe any assumptions made about any missing or unclear information.	Lines 854-860
Study risk of bias assessment	11	Specify the methods used to assess risk of bias in the included studies, including details of the tool(s) used, how many reviewers assessed each study and whether they worked independently, and if applicable, details of automation tools used in the process.	Lines 860-864
Effect measures	12	Specify for each outcome the effect measure(s) (e.g. risk ratio, mean difference) used in the synthesis or presentation of results.	N/A
Synthesis methods	13a	Describe the processes used to decide which studies were eligible for each synthesis (e.g. tabulating the study intervention characteristics and comparing against the planned groups for each synthesis (item #5)).	Lines 815-819, 868-867
	13b	Describe any methods required to prepare the data for presentation or synthesis, such as handling of missing summary statistics, or data conversions.	Lines 836, 839-844

Section and Topic	Item #	Checklist item	Location where item is reported
	13c	Describe any methods used to tabulate or visually display results of individual studies and syntheses.	Lines 868-871
	13d	Describe any methods used to synthesize results and provide a rationale for the choice(s). If meta-analysis was performed, describe the model(s), method(s) to identify the presence and extent of statistical heterogeneity, and software package(s) used.	Lines 875-884
	13e	Describe any methods used to explore possible causes of heterogeneity among study results (e.g. subgroup analysis, meta-regression).	N/A
	13f	Describe any sensitivity analyses conducted to assess robustness of the synthesized results.	N/A
Reporting bias assessment	14	Describe any methods used to assess risk of bias due to missing results in a synthesis (arising from reporting biases).	N/A
Certainty assessment	15	Describe any methods used to assess certainty (or confidence) in the body of evidence for an outcome.	Lines 887-895
**RESULTS**			
Study selection	16a	Describe the results of the search and selection process, from the number of records identified in the search to the number of studies included in the review, ideally using a flow diagram.	Lines 143-178
	16b	Cite studies that might appear to meet the inclusion criteria, but which were excluded, and explain why they were excluded.	N/A
Study characteristics	17	Cite each included study and present its characteristics.	Line 182-622
Risk of bias in studies	18	Present assessments of risk of bias for each included study.	Lines 625-626, Table S1
Results of individual studies	19	For all outcomes, present, for each study: (a) summary statistics for each group (where appropriate) and (b) an effect estimate and its precision (e.g. confidence/credible interval), ideally using structured tables or plots.	N/A
Results of syntheses	20a	For each synthesis, briefly summarise the characteristics and risk of bias among contributing studies.	Lines 234-238, 311-314. 357-360, 396-402, 424-426, 486-487, 523-526, 556-557, 594-596, 606-608, 621-623. Table 6
	20b	Present results of all statistical syntheses conducted. If meta-analysis was done, present for each the summary estimate and its precision (e.g. confidence/credible interval) and measures of statistical heterogeneity. If comparing groups, describe the direction of the effect.	N/A
	20c	Present results of all investigations of possible causes of heterogeneity among study results.	N/A
	20d	Present results of all sensitivity analyses conducted to assess the robustness of the synthesized results.	N/A

Section and Topic	Item #	Checklist item	Location where item is reported
Reporting biases	21	Present assessments of risk of bias due to missing results (arising from reporting biases) for each synthesis assessed.	N/A
Certainty of evidence	22	Present assessments of certainty (or confidence) in the body of evidence for each outcome assessed.	Table 6
**DISCUSSION**			
Discussion	23a	Provide a general interpretation of the results in the context of other evidence.	Lines 640-707
	23b	Discuss any limitations of the evidence included in the review.	Lines 640-707
	23c	Discuss any limitations of the review processes used.	Lines 709-743
	23d	Discuss implications of the results for practice, policy, and future research.	Lines 746-760
**OTHER INFORMATION**			
Registration and protocol	24a	Provide registration information for the review, including register name and registration number, or state that the review was not registered.	Lines 768-779
	24b	Indicate where the review protocol can be accessed, or state that a protocol was not prepared.	Lines 768-779
	24c	Describe and explain any amendments to information provided at registration or in the protocol.	Lines 768-779
Support	25	Describe sources of financial or non-financial support for the review, and the role of the funders or sponsors in the review.	Lines 905-910
Competing interests	26	Declare any competing interests of review authors.	Lines 914-916
Availability of data, code and other materials	27	Report which of the following are publicly available and where they can be found: template data collection forms; data extracted from included studies; data used for all analyses; analytic code; any other materials used in the review.	Lines 912-913

Table S4. Database search strategy MEDLINE(PubMed)
#1. cefazolin[ALL] OR cefazoline[ALL] OR cephazolin[ALL]
#2. cefazolin[MH] OR cephalosporins[Mesh:noexp]
#3. #1 OR #2
#4. surger*[TIAB] OR surgical*[TIAB] OR perioperative[TIAB] OR preoperative[TIAB] OR incision[TIAB] OR "antibiotic prophylaxis"[TIAB]
#5. antibiotic prophylaxis[MH]
#6. #4 OR #5
#7. concentration*[TIAB] OR level*[TIAB] OR absorption[TIAB] OR penetration[TIAB] OR clearance*[TIAB] OR pharmacokinetic*[TIAB] OR pharmacodynamic*[TIAB] OR "minimum inhibitory concentration"[TIAB] OR "MCI"[TIAB]
#8. tissue*[TIAB] OR serum*[TIAB] OR bone*[TIAB] OR blood*[TIAB] OR plasma[TIAB] OR "interstitial fluid"[TIAB] OR "extracellular fluid"[TIAB] OR biops*[TIAB] OR sample*[TIAB] OR sampling[TIAB] OR test*[TIAB]
#9. #7 AND #8
#10. adolescent[MH] OR child[MH] OR infant[MH] OR animal experimentation[MH] OR models, animal[MH] OR animals[Mesh:noexp]
#11. child*[TI] OR adolescent*[TI] OR pediatric*[TI] OR peadiatric*[TI] OR infant*[TI] OR neonat*[TI] OR newborn*[TI] OR porcine[TI]
#12. #10 OR #11
#13. #3 AND #6 AND #9 NOT #12
**Year Limit:** nil
**PubMed:** Medline
All fields [ALL], Title [TI], Title and Abstract [TIAB], MeSH Term [MH], MeSH Term Unexploded [Mesh:noexp]

Table S5. Database search strategy EMBASE
#1. cefazolin:ti,ab OR cefazoline:ti,ab OR cephazolin:ti,ab
#2. 'cefazolin'/exp OR 'cephalosporin derivative'/de
#3. #1 AND #2
#4. surger*:ti,ab OR surgical*:ti,ab OR perioperative:ti,ab OR preoperative:ti,ab OR incision:ti,ab OR “antibiotic prophylaxis”:ti,ab
#5. 'antibiotic prophylaxis'/exp
#6. #4 OR #5
#7. concentration*:ti,ab OR level*:ti,ab OR absorption:ti,ab OR penetration:ti,ab OR clearance*:ti,ab OR pharmacokinetic*:ti,ab OR pharmacodynamic*:ti,ab OR 'minimum inhibitory concentration':ti,ab OR “MCI”:ti,ab
#8. tissue*:ti,ab OR serum*:ti,ab OR bone*:ti,ab OR blood*:ti,ab OR plasma:ti,ab OR 'interstitial fluid':ti,ab OR 'extracellular fluid':ti,ab OR biops*:ti,ab OR sample*:ti,ab OR sampling:ti,ab OR test*:ti,ab
#9. #7 AND #8
#10. juvenile/exp OR 'animal experiment'/exp OR 'animal model'/exp OR animal/de
#11. child*:ti OR adolescent*:ti OR pediatric*:ti OR peadiatric*:ti OR infant*:ti OR neonat*:ti OR newborn*:ti OR porcine:ti
#12. #10 OR #11
#13. #3 AND #6 AND #9 NOT #12
**Year Limit:** nil
Article title :ti, abstract :ab, index term /de, drug trade name :tn, exploded index term /exp.

Table S6. CKN EBSCO EDS discovery layer additional databases.
•Academic Search Index
•AccessScience
•BioOne Complete
•BMJ Best Practice

- BMJ Clinical Evidence
- CINAHL Complete
- ClinicalTrials.gov
- Cochrane Database of Systematic Reviews
- CogPrints
- Complementary Index
- Directory of Open Access Journals
- Emerald Insight
- ERIC
- Harvard Library Bibliographic Database
- Informit Health Collection
- Informit Indigenous Collection
- McGraw-Hill Medical
- MEDLINE Complete
- Minority Health Archive
- Oxford Medicine Online
- Oxford Reference
- R2 Digital Library
- Rural and Remote Health Database
- ScienceDirect
